# A novel isoform of IL-33 revealed by screening for transposable element promoted genes in human colorectal cancer

**DOI:** 10.1371/journal.pone.0180659

**Published:** 2017-07-17

**Authors:** Frances E. Lock, Artem Babaian, Ying Zhang, Liane Gagnier, Sabrina Kuah, Antonia Weberling, Mohammad M. Karimi, Dixie L. Mager

**Affiliations:** 1 Terry Fox Laboratory, British Columbia Cancer Agency, Vancouver, BC, Canada; 2 Department of Medical Genetics, University of British Columbia, Vancouver, BC, Canada; 3 Département de Génomique Fonctionnelle et Cancer, Institut de Génétique et Biologie Moléculaire et Cellulaire (IGBMC)/Université de Strasbourg/CNRS/INSERM, France; University of Muenster, GERMANY

## Abstract

Remnants of ancient transposable elements (TEs) are abundant in mammalian genomes. These sequences contain multiple regulatory motifs and hence are capable of influencing expression of host genes. TEs are known to be released from epigenetic repression and can become transcriptionally active in cancer. Such activation could also lead to lineage-inappropriate activation of oncogenes, as previously described in lymphomas. However, there are few reports of this mechanism occurring in non-blood cancers. Here, we re-analyzed whole transcriptome data from a large cohort of patients with colon cancer, compared to matched normal colon control samples, to detect genes or transcripts ectopically expressed through activation of TE promoters. Among many such transcripts, we identified six where the affected gene has described role in cancer and where the TE-driven gene mRNA is expressed in primary colon cancer, but not normal matched tissue, and confirmed expression in colon cancer-derived cell lines. We further characterized a TE-gene chimeric transcript involving the Interleukin 33 *(IL-33*) gene (termed LTR-*IL-33*), that is ectopically expressed in a subset of colon cancer samples through the use of an endogenous retroviral long terminal repeat (LTR) promoter of the MSTD family. The LTR-*IL-33* chimeric transcript encodes a novel shorter isoform of the protein, which is missing the initial N-terminus (including many conserved residues) of Native IL-33. *In vitro* studies showed that LTR-IL-33 expression is required for optimal CRC cell line growth as 3D colonospheres. Taken together, these data demonstrate the significance of TEs as regulators of aberrant gene expression in colon cancer.

## Introduction

Gene deregulation is widespread in cancer and can be due to mutations in cis regulatory motifs, disruptions in epigenetic state or dysregulation of other regulatory pathways. One underappreciated mechanism that can cause gene deregulation in cancer is activation of cryptic (or normally dormant) promoters [1–4]. Transposable elements (TEs), including endogenous retroviruses (ERVs) and long interspersed elements (LINEs), comprise nearly half of the human genome [5–7] and represent an abundant source of natural promoters in the genome [8, 9]. In particular, ERV long terminal repeats (LTRs), the termini of integrated retroviruses, naturally harbor promoters and enhancers. Therefore, the >400,000 ERV LTRs in the genome have built-in potential to affect gene expression, as has been shown by many studies [8, 10–18].

While ERV and LINE L1 transcription is generally suppressed in normal cells by epigenetic mechanisms and multiple host factors [19–26], hypomethylation and transcriptional up-regulation of ERVs and L1s is often observed in cancers [27–31], likely a reflection of widespread epigenetic dysregulation [32–34]. Such deregulation could contribute to somatic L1 retrotransposition events that have been documented in several human tumor types [35–41]. In mouse, ERV insertions can activate oncogenes [42, 43] but human ERVs are older and unable to retrotranspose [44]. However, it is possible that existing ERVs or L1s, normally dormant, could become transcriptionally activated and drive oncogenic gene expression. Indeed, in recent years a growing number of genes and long non-coding (lnc) RNAs with oncogenic/growth promoting effects have been shown to be ectopically expressed from TEs (mainly ERV LTRs) [3, 45–56], a process we have termed “onco-exaptation” [54, 57].

While these reports have spurred interest, the overall prevalence and significance of TE-driven aberrant gene expression in cancer is underreported, and has not been assessed in colon cancer. We hypothesize that cancer-associated release of epigenetic suppression of TEs could result in significant perturbations to the transcriptome, some of which could play a role in carcinogenesis.

Over 1.4 million new cases of CRC are diagnosed per year worldwide [58, 59], and there are limited treatment options and high mortality for patients with metastasis [60]. CRC is a heterogeneous disease and its development is influenced by multiple environmental and genetic factors [61]. While much is known of mutations, epigenetic and expression perturbations in CRC [62–66], less is known of mechanisms resulting in aberrant gene regulation. Here, we applied a novel bioinformatics pipeline, *LIONS*, to published [66] RNA-seq data from 66 primary colorectal cancer (CRC) samples and matched normal colon from the same individual to comprehensively identify TE-driven transcripts specific to, or enriched in, the cancer samples. This led to the identification of many cancer-enriched and recurrently arising TE-driven transcripts in primary CRC. Candidate transcripts were validated in CRC cell lines and we focused on one particular such case, which produces a novel N-terminal truncated isoform of Interleukin-33 (IL-33).

IL-33 plays an important role in chronic inflammation such as in inflammatory bowel disease [67–69]. Given the importance of inflammation in cancer, recent studies have begun to investigate the role of IL-33 and its receptor ST2 (also termed IL1R1) in CRC [70]. Indeed, IL-33 is elevated in the serum of patients with lung, gastric and hepatocellular cancer [71] and is a marker for poor prognosis [11, 69]. Previous work showed that higher expression of total IL-33 and ST2 correlates with CRC progression and metastasis, with inhibition of IL-33 in CRC cells resulting in reduced cell migration, colony formation and tumor growth in vitro, and smaller tumors in vivo [72, 73]. Moreover, using patient derived primary CRC cell lines as well as a mouse model of CRC, IL-33 was also suggested to activate colon tumor stroma and promote polyposis in vivo [74]. Given these previous studies, here we sought to further characterize the LTR-promoted isoform of *IL-33* and investigate its potential role in CRC.

## Materials and methods

All reagents were purchased from Sigma (Ontario, Canada), unless specified. All experiments are representative of at least 3 independent experiments, unless specified. Statistical analysis was performed using T test, unless specified. See S1 Table for all primers used in this study.

### Bioinformatics analysis

To comprehensively examine and quantify TE promoter activation in cancer, we developed a bioinformatics pipeline called *LIONS*, to mine RNA-seq data for detection of TE-initiated transcripts [75] and applied it to an RNA-seq dataset of CRC samples and matched normal colon [66]. Briefly, each paired-end RNA-seq library was aligned to the human reference genome *hg19* with *tophat2* [76] and a transcriptome assembled *ab initio* with *Cufflinks2* [77]. Assembled-contig 5’ ends as well as clusters of read-pairs extending the contigs were analyzed for overlap with annotated TEs to define a set of TE-initiated transcripts.

*LIONS* was run with two parameter sets; ‘relaxed’ to maximize sensitivity or ‘stringent’ to maximize specificity. Parameters were *‘—scREADS 3 –scTHREAD 5 –scRPKM 1 –scCONTR 0*.*1 –scUPCOV 2 –scUPEXON 1*.*5 –spCOE <library_size_in_reads>/20000000’* for relaxed mode and *‘—scREADS 6 –scTHREAD 10 –scRPKM 1 –scCONTR 0*.*5 –scUPCOV 3 –scUPEXON 1*.*5 –spCOE <library_size_in_reads>/10000000’* for stringent mode. This means that with stringent thresholds, to call a TE-initiated transcript; 6 independent read-pairs are necessary; within the TE-boundary, read-pairs should have a 10-fold bias in the direction of transcription emanating from them; the gene exon should be expressed to a minimum Reads Per Kilobase of transcript per Million mapped reads (RPKM) of 1; the TE-isoform must contribute at least 50% of the total gene’s expression; sequence coverage within the TE should be 3-fold higher than adjacent, upstream sequence; and if applicable upstream genic exons should be expressed less than 1.5-fold of the TE-exon pair. The number of supporting read-pairs required was 3 or 6. Due to “transcriptional noise”, a large proportion of TE-initiated transcripts contribute little to a gene’s overall expression, thus TE-initiated transcripts contributing less than 10% (relaxed thresholds) or less than 50% (stringent thresholds) of a gene’s overall expression in a given library were filtered out. Sets of TE-initiated transcripts were then compared.

To account for differing sequencing depths, a chimeric fragment cluster upon which a TE-initiation is called requires a threshold number of supporting reads. That threshold is dependent on library size, for 'relaxed' criteria it is the greater of 3 or 1/20 million reads in library, for 'stringent' criteria it is the greater of 6 or 1/10 millions reads in library.

### 5′ RACE

To confirm sequence of chimeric 5′ ends, 5′ RACE was performed on 1 μg HT115 RNA using a First Choice RLM-RACE kit (Ambion) as per the manufacturer’s protocol. PCR to amplify 5′ ends of gene of interest was performed using primers supplied and gene-specific primers IL-33 RACE-AS1 for the first round and IL-33 RACE-AS2 for the second round. (See S1 Table for all primers). Amplification was performed with Bestaq DNA polymerase (Applied Biological Materials Inc.) at 60°C annealing during the first round and 63°C annealing during the second round, 20-s elongation, and 35 amplification cycles. PCR-amplified 5′ end transcripts were cloned into Promega pGEM T vector (Promega) and sequenced by Eurofins MWG Operon.

### RT-PCR of full length LTR-IL-33 ORF

HT115 RNA was extracted (two biological replicates) with the All Prep DNA/RNA mini kit (Qiagen) according to the manufacturer’s instructions. Five hundred nanograms of DNase-treated (Ambion Turbo DNase) RNA was used for cDNA synthesis with Vilo reverse transcriptase (Invitrogen). All primers used for cDNA amplification encompass at least one intron to check for genomic DNA contamination. Primers IL-33_LTR_F and IL-33_tot_R were used to amplify 1 μg of cDNA in a 25-μL reaction, using Bestaq DNA polymerase (Applied Biological Materials Inc.) at 64°C annealing, 30-s elongation, and 35 amplification cycles. Transcripts were cloned into Promega pGEM T vector (Promega) and sequenced by Eurofins MWG Operon.

### Cloning of IL-33 into the Flag-tagged expression vector

The native and LTR forms of IL-33 were amplified separately from LoVo cDNA using Phusion DNA polymerase (New England Biolabs) at 63°C annealing, 1 min elongation, and 35 amplification cycles using primers IL33native EcoRI and IL33ex8 XhoI-2 for the native form and IL33LTR EcoRI and IL33ex8 XhoI-2 for the LTR form. Forward and reverse primers contained recognition sites specifically designed for EcoRI and XhoI respectively. After digestion with EcoRI and XhoI and purification, the fragments were cloned into the EcoRI and XhoI sites of pCMV-3Tag3 expression vector (Sigma) and sequenced by Eurofins MWG Operon.

### Bisulfite analysis

Bisulfite conversion, semi- nested PCR, cloning, and sequencing were carried out as described previously [78]. All of the sequences included in the analyses either displayed unique methylation patterns or unique C-to-T non-conversion errors (remaining Cs not belonging to a CpG di-nucleotide) after bisulfite treatment of the genomic DNA. This avoids considering several PCR-amplified sequences from the same template molecule (provided by a single cell). All sequences had a conversion rate >95%. Primers used in the first round for the LTR form were IL-33 LTRBIS-S3 and IL-33 LTRBIS-AS3 and for the second round IL-33 LTRBIS-S4 and IL-33 LTRBIS-AS3. First round primers for the native form were IL-33 natBIS-S1 and IL-33 natBIS-AS1 and for the second round IL-33 natBIS-S2 and IL-33 natBIS-AS1. Sequences were analyzed with QUMA free online software [79].

### RT-PCR, qRT-PCR and Western blotting

For RT-PCR, RNA was extracted (at least two biological replicates per cell line) with the All Prep DNA/RNA mini kit (Qiagen). 1μg DNAse treated (Ambion Turbo DNAse) RNA was used for cDNA synthesis with SuperScript III (Invitrogen). All primers used for cDNA amplification encompass at least one intron to check for genomic DNA contamination, as described previously [56]. qRT-PCR was performed using Applied Biosystems Fast SYBR master mix on an ABI7500 Fast system with normal cycling parameters. Expression levels were normalized to a control gene, β-actin, by the ΔΔCT method. For western blotting, cells were lysed with RIPA buffer with protease inhibitors. 40 μg of protein per sample were separated using 4–12% Bis-Tris gels and proteins transferred to PVDF membrane using Tris-glycine transfer buffer (Invitrogen, Carlsbad, CA, USA), blocked with milk-TBST (Tris-buffered saline with 0.5% Tween-20) and stained with antibodies specific to the target protein, as appropriate: Flag, #14793, alpha / beta tubulin #2148, Cell Signaling Technology, MA; Rabbit IgG control, Millipore, CA; Actin, A2066, GAPDH, Sigma; IL-33, (Nessy-1) ALX-804-840 Enzo Life Sciences, NY; PCNA, 610664, BD Transduction labs, San Jose, CA; Lamin A, Ab8980, AbCam, Toronto, Canada; anti-rabbit-HRP, anti-mouse-HRP (Sigma).

### Nuclear Cytosol separation and lysis

Nuclear and cytoplasmic fractionation was performed by a modification of the method described previously [80]. Briefly, cells were scraped into cold PBS, and lysed on ice for 15 min in 100 ml of cytoplasmic lysis buffer (10 mM HEPES, pH 7.4, 10 nM KCl, 0.01 mM EDTA, 0.1 mM EGTA, 2 mM dithiothreitol, 5 mM Na2VO4, 20 mM sodium β-glycerophosphate, 0.1% Nonidet P-40 and protease inhibitor cocktail (Roche)). Nuclei were sedimented by centrifugation, and the supernatant containing the cytoplasmic fraction was removed. Urea and SDS were added to a final concentration of 2 M and 2% respectively, and the samples were denatured by boiling for 5 min. The nuclei were then lysed in sample buffer.

### Site-directed mutagenesis

Two of the 3 original in-frame ATGs (Methionine, MMM) of the LTR-IL-33 isoform were systematically changed to ATA (Isoleucine, resulting in MII, IIM and IMI) A negative control with all 3 ATGs changed to ATAs was also created (III). Point mutations were generated in the pCMV3Tag3–LTR-IL-33 construct using the QuikChange Lightning site-directed mutagenesis kit (Agilent) as per the manufacturer’s instructions. 50 ng of pCMV3Tag3 –LTR-IL-33 plasmid was used as a template, with primers g152a and g152a_antisense to create IMM, with primers g161a and g161a_antisense to create MIM, with primers g182a and g182a_antisense to create MMI and primers g152a_g161a and g152a_g161a_antisense to create IIM. IMM, MIM and IIM constructs were subjected to another round of site directed mutagenesis using primers g182a and g182a_antisense to create IMI, MII and III. The QuikChange reaction products were treated with *Dpn*I at 37°C for 10 min to deplete the original plasmid. 5 μL was transformed into XL10 Gold cell and transformants were sequenced by Eurofins MWG Operon.

### Chromatin immunoprecipitation

ChIP was carried out as described previously [81], with some modifications. Briefly, 293T HEK cells were transfected to exogenously express Flag-tagged Native IL-33, LTR-IL-33 or the empty vector pCMV3Tag3. 10^6^ cells were lysed for Western blotting to confirm exogenous protein expression. Cells were processed to extract gDNA, and incubated with anti-Flag antibody or a matched IgG control. qPCR analysis was carried out using primers against the p65 promoter region, or an unrelated region, as described [82], or against the ST2 distal or proximal promoter region, or an unrelated region, as described [83]. Data is presented as % input chromatin signal. Results shown are representative of two independent experiments.

### siRNA transfections

Where appropriate, cell lines were transfected at subconfluence with Non-Silencing Control siRNA (#12935–300, Invitrogen, CA), or two disparate siRNA targeting IL-33 (siRNA1 (131654), siRNA2 (131665), (#AM16708, Ambion, CA) using Lipofectamine 2000, according to manufacturer’s instructions. After attaining confluence (3 days), cells were passaged, and the transfection repeated. Once cells again attained confluence, cells were processed as required.

### Luciferase assay

The p65 promoter fragment of approximately 1300bp was amplified from HUVEC gDNA using Phusion DNA polymerase (New England Biolabs) at 64°C annealing, 40-s elongation, and 35 amplification cycles using primers p65 prom KpnI-s and p65 prom XhoI-as. Forward and reverse primers contained recognition sites specifically designed for the KpnI and XhoI respectively. After TA mediated cloning into T-vector (Promega) the fragment was cut out with KpnI and XhoI and purified, the fragment was cloned into the KpnI and XhoI sites of pGL4.10 [*luc2*] promoterless vector (Promega) and sequenced by Eurofins MWG Operon. Appropriate Cell lines were transiently transfected with either pCMV3Tag3-empty vector, Native IL-33-pCMV3Tag3 or LTR-IL-33-pCMV3Tag3 in combination with pGL4.10 empty vector or p65 promoter-pGL4 and the renilla transfection control plasmid pRL TK. After 43h, promoter activity, measured as relative luciferase units (Firefly / Renilla) was assessed using dual-reporter assay kit Stop ‘N’ Glow (Promega). Exogenous expression of Flag-tagged Native IL-33 or LTR-IL-33 was confirmed by western blotting for each experiment.

The MSTD LTR promoter fragments of 502 (long) and 316 bp (short) were amplified separately from LS513 gDNA using Bestaq DNA polymerase (Applied Biological Materials Inc.) at 62°C annealing, 20-s elongation, and 35 amplification cycles. As this is a very repeat rich region nested PCR (primers IL33 LTR-s-NheI / IL33 LTR as2-XhoI) was required. Forward and reverse primers (IL33 LTR s2-NheI / IL33 LTR-as3-XhoI /IL33 LTR-s3-Nhe1) contained recognition sites specifically designed for the NheI and XhoI respectively. After digestion with NheI and XhoI and purification, the fragment was cloned into the NheI and XhoI sites of pGL4.10 [luc2] promoterless vector (Promega). The intermediate region was cloned from the long region using an existing BglII site which removed the first 107 base pairs. This intermediate region was then blunt end cloned into pGL4.10. All constructs were sequenced by Eurofins MWG Operon before use.

### Cell culture and transfections

Cells were maintained at 37°C, 5% CO_2_ in a humidified atmosphere. Unless, specified, culture reagents were purchased from Gibco. 293T HEK were cultured DMEM (StemCell Technologies, Vancouver, BC) with 10% FBS, 1% L-Glutamine. 293T HEK cells were transfected using CaPO4 transfection. LS513 cells (a kind gift from Dr. M Lacroix, at the INRS-Institut Armand-Frappier, QC) cultured in RPMI (Stem Cell Technologies, Vancouver, BC), 10% FBS, 1% sodium pyruvate, 1% non-essential amino acids); RKO cells were cultured in Eagle's Minimum Essential Medium, 10% Donor Calf Serum; CaCo2 cells were cultured in EMEM, 10% FBS; HCT116, LoVo, SW620, SW480, WiDr, MIP101 cells were cultured in DMEM, 10% FBS; HT115 cells (a kind gift from Dr. C Guillemette, Laval University, QC) were cultured in DMEM, 2mM Glutamine, 15% FBS; HT29, Colo205 cells were cultured in RPMI1640, 10% FBS. Where appropriate, cells were transfected using Lipofectamine 2000 transfection reagent following manufacturers protocol. HUVEC (Lonza) cultured in EGM-2 Bullet kit media (Lonza) +1% PenStrep according to manufacturer instructions.

For the generation of 3D colonospheres, 200 cells were plated per well, on ultra-low attachment 96-well plates (Corning, NY), then subjected to 7 doubling dilutions (in quadruplicate) and grown in colonosphere medium (DMEM, F12þGlutMAX-I (Gibco), 1% N2 (Gibco), 2% B27 (Gibco), 20 ng/ml hFGF-2 (Sigma, MO), 50 ng/ml EGF (Sigma). After 7 days, plates were analyzed for colonosphere formation, as previously described [84].

### Media collection and precipitation

Cells were cultured to confluence under standard conditions. At confluence, media was removed, cells were washed with PBS, and replaced with serum-free culture media. The confluent monolayer was then repeatedly scratched with a sterile 1ml pipette tip to create a “wound”, or left unwounded, as a control. Cells were cultured for 24h. Conditioned media was then removed, centrifuged at 4500g to remove cellular debris and the supernatant subjected to TCA precipitation, as described previously [85]. Precipitates were solubilised in SDS PAGE sample buffer, boiled and subjected to Western blotting.

## Results and discussion

### Prevalence of TE-initiated chimeric transcripts in CRC and matched normal datasets

**Fig 1:**
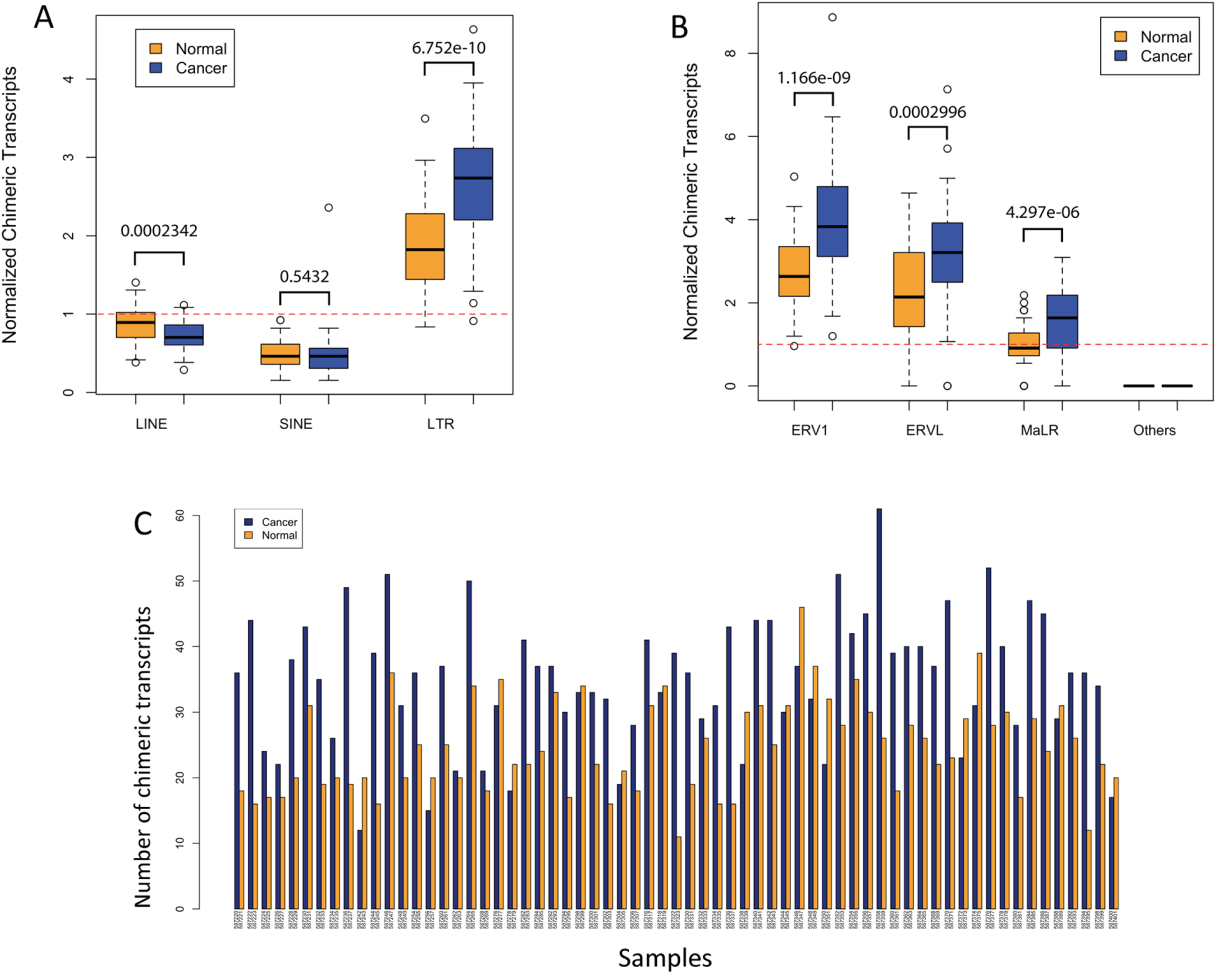
A) Comparison of numbers of TE-initiated chimeric transcripts between normal and cancer samples based on stringent thresholds (see Methods). The total number of such transcripts of each TE class was adjusted by their genomic coverage, and also normalized by the expected expression based on all chimeric transcripts in normal samples (the red dotted line). The box plot shows an interquartile range of 50% for each sample group, and outlier samples are shown when the numbers of chimeric transcripts are beyond one interquartile range from the edge of box. P-values are based on T-test. B) Similar plot for the three major ERV classes. C) Total numbers of LTR-initiated chimeric transcripts between normal and cancer samples of each individual patient based on stringent thresholds. The cancer and normal sample pair of each individual is shown as side-by-side bars in blue and orange, respectively. The height of the bars shows the total number of chimeras in each sample corrected by the library size.

Using the criteria and thresholds described in Materials and Methods, we screened the RNA-seq datasets from 66 CRC samples and matched normal colon [66] for TE-initiated transcripts and the full results are shown in S2 Table (both “relaxed” and “stringent” lists). To determine which class of TEs contributed most to promoting chimeric transcripts, we plotted the relative numbers of such transcripts normalized by genomic abundance (total genomic coverage of each TE class). Fig 1A and S1A Fig show that for both the cancer and normal samples, LTR-promoted chimeric transcripts are over-represented and LINE and SINE promoted chimeric transcripts are underrepresented based on genomic abundance. Such a result is expected since the vast majority of ERV/LTR related sequences in the genome are in the form of solitary LTRs, which naturally contain promoters [18, 86, 87]. In contrast, most LINE-related sequences in the genome are 5’ truncated, lacking the promoter [88, 89], and SINE sequences such as Alu elements contain PolIII promoters, which are weak and position dependent [90, 91]. Notably, the average number of LTR-promoted transcripts is significantly higher in the cancer samples, possibly indicating widespread de-repression of LTRs in colon cancer. This trend holds for all major classes of human ERVs, namely LTRs of the ERV1, ERVL and MaLR classes, with the latter two being generally older than ERV1 elements [87, 92] (Fig 1B and S1B Fig).

Fig 1C and S1C Fig show the actual numbers of LTR-initiated chimeric transcripts in each cancer/normal pair for the stringent and relaxed criteria, respectively. While there is significant variability among samples, 73.8% of matched sample pairs show more LTR-initiated transcripts in the cancer samples than in controls (as assessed by exact binomial test (P-value = 0.0001521), again suggesting de-repression of LTRs in this malignancy.

### Recurrent chimeric transcripts

TEs, particularly LTRs, have been shown by many studies to promote gene transcription in both normal and cancer cells [18, 93, 94]. In this data set, transcripts statistically more recurrent (enriched) in the cancer or normal samples are listed in S3 and S4 Tables, respectively.

In this study, we focused on “cancer-enriched” chimeric transcripts or those transcripts found only in the cancer samples, even if their recurrence did not reach statistical significance, as these represent potential gain-of-function events that could play roles in the malignancy. For molecular validation, we chose the chimeric transcripts involving six genes or lncRNAs for which a role has been previously reported in cancer, which were recurrent in the cancer samples and which were not present in any normal samples. As well, for all six cases, the TE promoter “contribution to expression” compared to the native promoter was high (above 50%). The six genes are listed in Table 1. *SLCO1B3* and *IL-33* are described below and in S2 Fig, and the others in S1 Text and S3–S5 Figs. We used RT-PCR or qRT-PCR (for increased sensitivity) to test for the presence of these chimeric transcripts in a panel of 12 CRC cell lines with the results summarized in Table 1, discussed below for IL-33 (see also S6B Fig) and shown in S6A Fig for the other genes. Positive cell lines were found for all cases, suggesting that the chimeric transcript forms are likely intrinsic to the malignant cells within the primary tumors. With the exception of LTR-IL-33 and SLCO1B3, all chimeric transcripts splice upstream of the native translational start site.

**Table 1:** Examples of cancer-specific, recurrent TE-promoted chimeric transcripts in colon cancer.

Gene*	Gene function/info	Reported role for gene in cancer type listed	TE promoter	Genomic coordinates of TE (hg 38)	TE contribution to expression^&^	# positive samples for chimera (out of 66) by RNA-seq^+^	# positive CRC cell lines for chimera (out of 12) by RT-PCR^@^
*SLCO1B3*	Ion transporter	Colon	LTR7	chr12:20822187–20822617	~100%	25	11
*IL-33*	cytokine	colon, others	MSTD LTR	chr9:6248332–6248575	Up to 100%	21	3^@^
*INPP4B*	PI3 signaling	Melanoma, colon, AML	L1PA14	chr4:142473171–142475297	70–100%	6	7
*ACTL8*	Testis antigen	Biomarker	LTR41	chr1:17755153–17755695	100% (annotated promoter)	6	8
*ST8SIA6-AS1*	lncRNA	Breast biomarker	MER48 LTR	chr10:17386607–17386994	100% (annotated promoter)	5	4
*MUCL1*	Mucin, HER2-responsive	breast	MER31 ERV	chr12:54830207–54833015	~100%	3	3

*Selected cases involving genes with known roles in cancer or as cancer biomarkers. For references see main text or S1 Text.

^+^Based on stringent threshold. None of the 66 matched normal colon samples had levels of the chimeric transcript passing the threshold.

^&^Estimated from RNA-seq read coverage over TE compared to native promoter in samples positive for the chimeric transcript.

^@^IL-33 transcripts were measured using quantitative RT-PCR.

#### SLCO1B3

**Fig 2:**
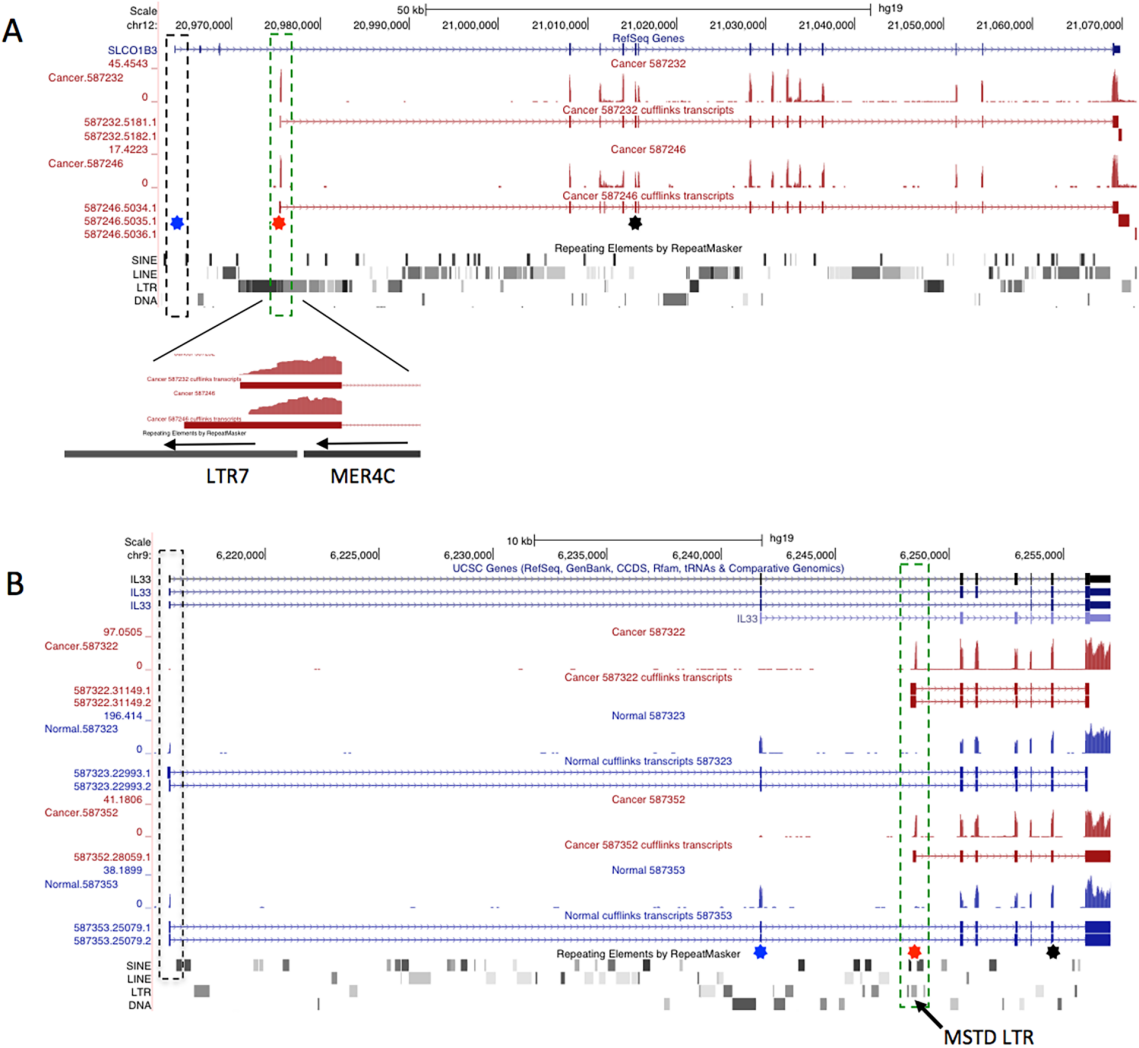
Representative UCSC genome browser views (hg19) of the *SLO1B3* and *IL-33* genes. A) RNA-seq coverage tracks and assembled transcripts for two CRC samples are shown below the RefSeq track for *SLO1B3*, with the Repeatmasker track below. Position of the normal first exon is boxed with a dashed black line. Position of the LTR derived first exon is shown with a green dashed box and a close-up of this region shown below. The transcript initiates in an antisense LTR7 and utilizes a splice donor site within an adjacent MER4C LTR. B) Similar view for the *IL-33* locus, showing two CRC samples (in red) and the matched normal samples (in blue). The MSTD LTR that initiates transcription in cancer samples is located in the 2nd intron and the position highlighted by a dashed green box. For both A and B, blue and red stars show locations of RT-PCR forward primers used to validate expression from the native or LTR promoter, respectively. The black star shows location of the common reverse primer.

Among the most recurrent chimeric transcripts is an LTR-driven isoform of the gene *SLCO1B3*, which encodes organic anion transporting polypeptide 1B3 (termed OATP1B3). This chimeric isoform, which produces a shorter protein lacking the first 28 amino acids, has been reported by several groups to be cancer-specific [50, 95] and is associated with poorer survival in CRC [51]. Our pipeline clearly identified the promoter for this isoform to be an antisense ERV LTR7 of the HERV-H group (Fig 2A), a fact not mentioned in a previous publication that mapped the transcriptional start site [50]. This case provides a good proof of principle for our screening method.

#### IL-33

IL-33 is a member of the IL1 family and is found in the nucleus and as a cell-free cytokine, where it signals through its receptor ST2 encoded by the *IL1R1* gene [9, 69, 96]. In mice, deletion of the N-terminal region responsible for nuclear localization results in constitutive release of IL-33 and lethal inflammation [97], indicating that nuclear retention is crucial for down-modulating the cytokine function of IL-33.

**Fig 3:**
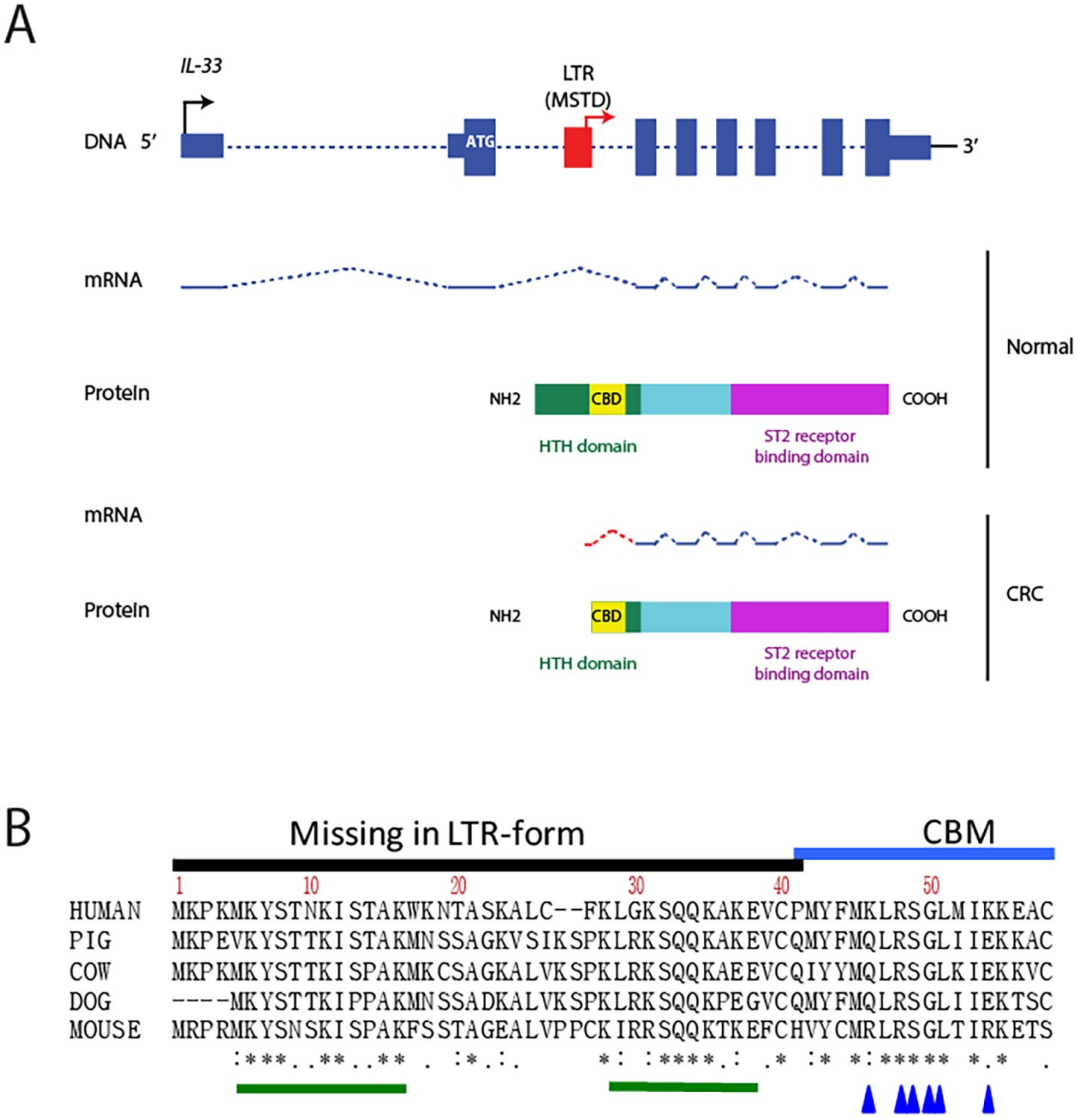
A) Schematic of the IL-33 gene and protein. Exons are depicted as blue boxes (DNA) and blue lines (mRNA). Introns are depicted as blue dashed lines. Thinner blue boxes are untranslated sequences and the normal ATG is depicted in white. MSTD-LTR is shown in red. Black and red arrows depict native and LTR transcription start sites, respectively. B) Amino acid alignment of the N-terminal homeodomain-like helix-turn-helix (HTH) region, including the chromatin binding motif (CBM) in IL-33 from different mammals. Asterisks show amino acid identity in all mammals, colons show near identity among mammals and single dots show conservative differences. Green bars show conserved motifs missing in the LTR form. Blue arrows show residues essential for chromatin association.

For 21 of 66 CRC RNA-seq libraries (but no matched normals), we found transcripts apparently initiating within an LTR of the MSTD family located in *IL-33* intron 2. Fig 2B shows representative samples and S2 Fig shows all CRC RNA-seq samples positive for this isoform. Since the normal ATG is in exon 2, the LTR-promoted form would lack the first 41 amino acids, if the first available in frame internal ATG is used for translation (Fig 3). Notably, sections of this N-terminal region are highly conserved among mammals (Fig 3B). Due to the fact that IL-33 has been shown to play a role in CRC and that the LTR-promoted form would theoretically produce a shorter, novel isoform of the protein, we focused on this gene for the rest of this study.

### Expression of LTR-IL-33 versus native expression in primary samples

**Fig 4:**
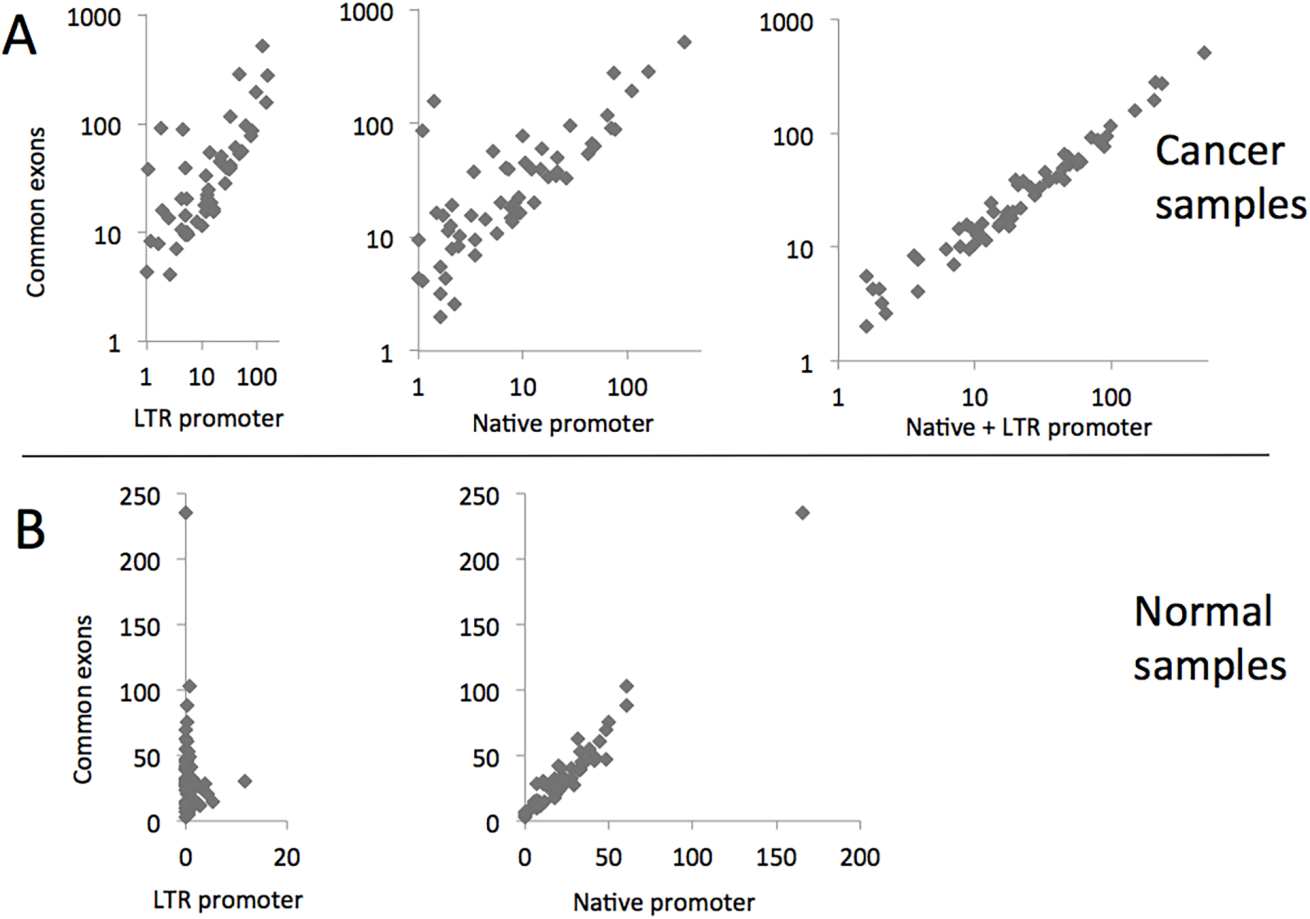
Contributions of the LTR and native promoter to IL-33 transcript levels. Peak RNA-Seq coverage over the LTR or the native promoter or a sum of the two is plotted relative to the peak coverage over downstream common exons 3 and 4, representing total expression. A) Cancer samples; B) Normal samples.

**Fig 5:**
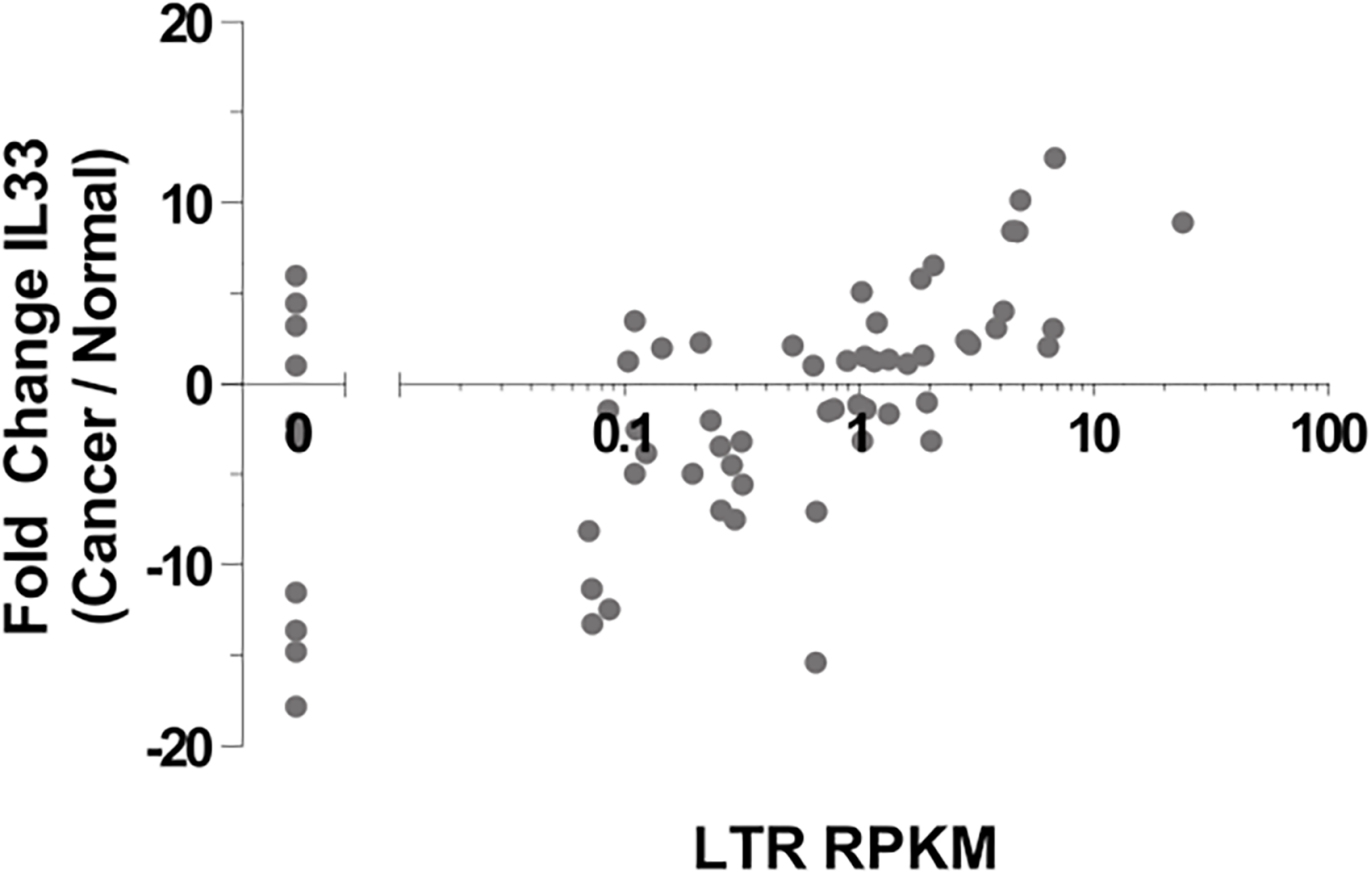
Difference in total *IL-33* expression (in RPKM) between matched normal-tumor pairs compared to the LTR-IL-33 exon RPKM in each cancer library. For each RNA-seq library, RPKM of each *IL-33* exon was calculated. Total gene expression was measured by the mean of exons 3–7, those common to both the native- and LTR-isoforms.

To assess relative contributions of the LTR and native promoter to overall *IL-33* transcript levels in the primary samples, we measured peak RNA-seq coverage over each promoter and over the next two common exons (canonical exons 3 and 4), as a measure of relative total expression. Interestingly, we found that both promoters make substantial contributions to overall transcript levels of *IL-33* in the primary CRC samples, and simply summing the values for the two promoters results in a tight correlation with total expression (Fig 4A). In contrast, the LTR promoter makes very little contribution to expression in the normal colon samples, with total transcription being essentially entirely accounted for by activity of the native promoter (Fig 4B). Within each sample, there is little correlation between activity of the LTR and native promoter (data not shown). Although some studies have reported higher overall *IL-33* expression in CRC compared to normal colon using probes/methods that would not have distinguished between the different promoters [72–74, 98], we did not observe a statistically significant difference between our RNA-seq datasets. However, there is a strong correlation between activity of the LTR promoter and the fold change in *IL-33* expression between matched cancer and normal samples. Namely, individuals in which the LTR is highly expressed in the cancer sample showed higher overall *IL-33* tumor expression compared to the matched normal colon (Spearman Correlation: r = 0.5925, P < 0.0001, ****) (Fig 5).

### Characterization of LTR-IL-33 transcription in cell lines

**Fig 6:**
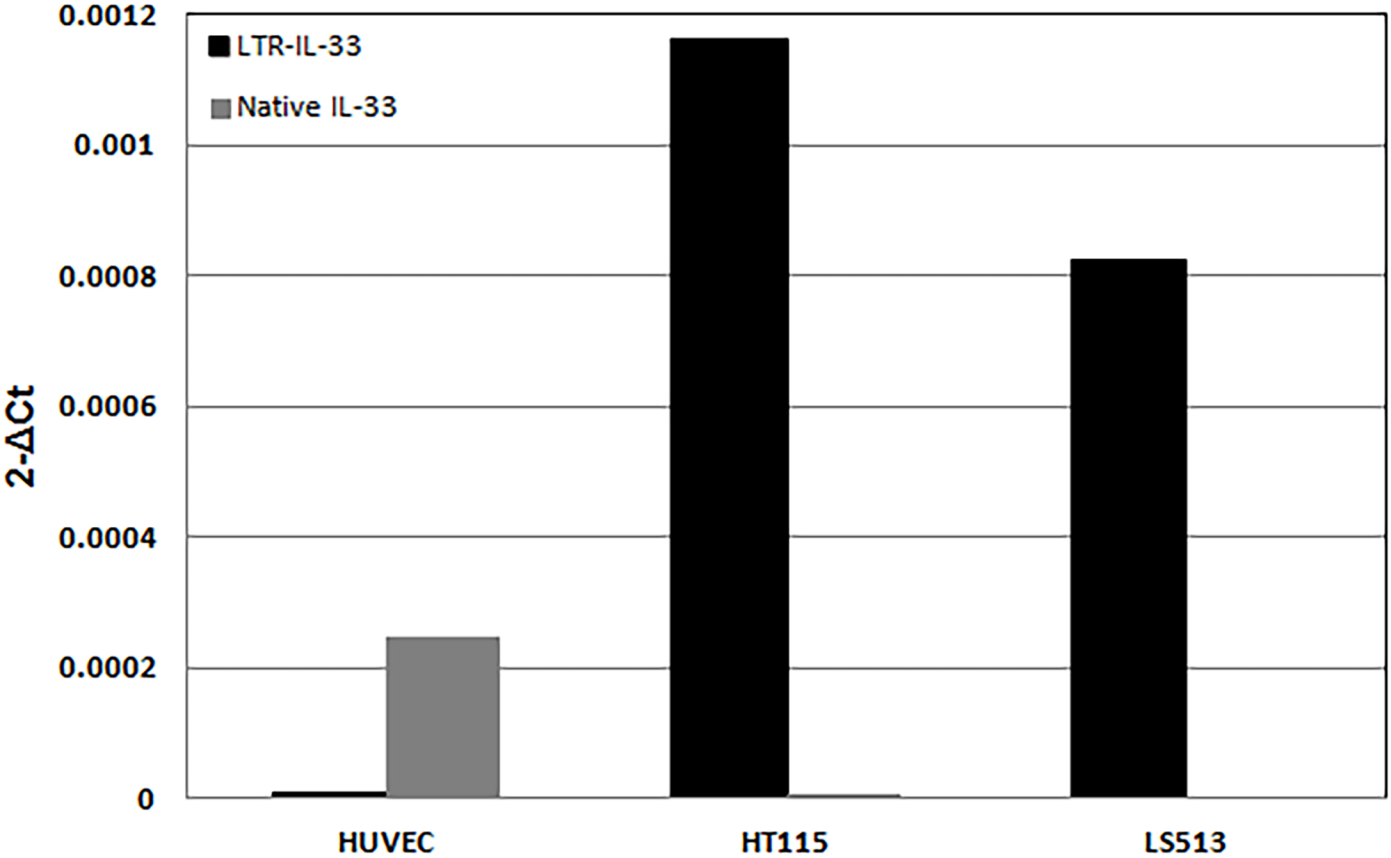
Quantitative RT-PCR analysis of native and LTR-IL-33 mRNA expression was carried out in HUVEC and colorectal cancer cell lines LS513 and HT115. Beta-actin levels were also assessed as an internal control.

Isoform-specific qRT-PCR assays found consistent expression of the LTR-IL-33 transcript in three CRC cell lines (S6B Fig), with highest expression in LS513 and HT115, neither of which showed significant expression from the native promoter (Fig 6, S6B Fig). Human umbilical vein endothelial cells (HUVEC), were null for LTR-IL-33 but express Native IL-33, which is upregulated at high cell confluence (as previously reported) [99]. Since chimeric LTR-*IL-33*, but no native transcript, was robustly amplified in the cell lines LS513 and HT115, they constitute good models to study the impact of TE-gene chimeras with limited interference from the native form.

To verify that the LTR-initiated transcript predicted from the RNA-Seq assembly is produced, we amplified and sequenced the full-length cDNA from HT115 cells and this is shown in S7 Fig. Alternate splicing of internal exons, reported in some cell lines [100], was not observed. Mapping of the LTR-driven transcriptional start site (TSS) in HT115 cells by 5’ RACE showed the major TSS to be located 35 bp downstream of a TATA box within the LTR (S8 Fig).

### The MSTD LTR region is able to function as a promoter

**Fig 7:**
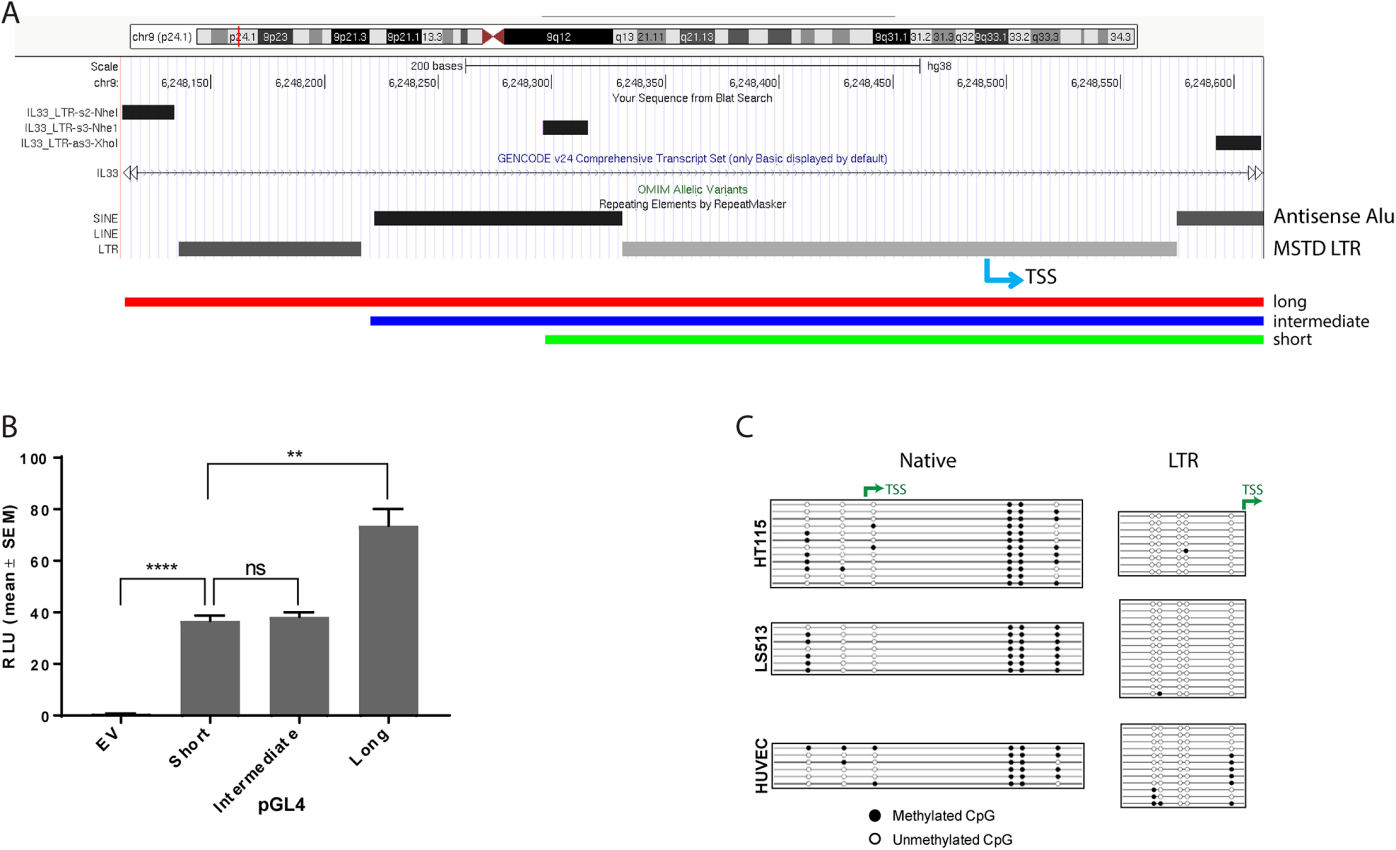
Promoter and methylation analysis. A) The MSTD LTR is in three sections, being interrupted by two antisense Alu elements. Part of the last Alu and the 3’ most LTR section are not shown. Long, intermediate and short sections of the MSTD LTR / Alu sequence were cloned into the pGL4 vector immediately upstream of the luciferase gene. Locations of primers are shown as bars at the top and also in S8 Fig. The LTR-IL-33 transcriptional start site (TSS) is also shown (blue arrow). (B) The “short”, “intermediate” and “long” luciferase constructs were transfected into LS513 cells and activity assessed by luciferase assay. Data from 2 independent experiments is shown. (C) Bisulfite sequencing of native and MSTD-LTR IL-33 promoters. The promoter regions of the LTR-IL-33-expressing cell lines HT115 and LS513, and the native IL-33-expressing cell line HUVEC were subjected to bisulphite analysis where CpGs were available. Filled circles represent methylated CpGs, empty circles represent unmethylated CpGs and each row represents an independent clone. The locations of transcription start sites (TSS) are shown with green arrows.

The intronic MSTD LTR is located between IL-33 exon 2 and 3 and is interrupted by two different antisense Alu insertions, a partial AluSc and a full length AluJr. UCSC Genome Browser primate comparisons indicate that the LTR and AluJr elements are present in New and Old World primates whereas the younger AluSc element is not present in New World monkeys. To verify that the LTR region can act as a functional promoter, we cloned a long, intermediate and short version of this region upstream of a luciferase reporter gene. The long version includes the most 5’ section of the LTR, the first Alu element and the middle section of the LTR containing the putative TATA box and TSS, whereas the short version just contains 35 bp of the first Alu and the middle LTR region. The intermediate version contains the first Alu and middle LTR section (Fig 7A and S8 Fig). These constructs were transfected into the colorectal cancer cell line LS513, which expresses endogenous LTR-IL-33. The short construct showed significant promoter activity, indicating that the middle LTR region, with possible contribution from the short Alu segment, is sufficient for promoter activity. Addition of the full first Alu element (intermediate) did not change promoter activity. However, the full length (long) MSTD sequence showed even greater promoter activity, indicating that motifs within the first and second LTR sections likely contribute to promoter activity (Fig 7B). Similar results were also observed in HT115 cells and also in two cell lines which do not endogenously express LTR-IL-33, namely 293T HEK and HCT116 (S9 Fig), suggesting that this region functions as a promoter in cell lines regardless of endogenous LTR-IL-33 expression.

### Activation of the LTR is not strongly correlated with DNA methylation state

Genome-wide hypomethylation, which affects TEs, along with localized hypermethylation of gene promoters, are well-known characteristics of cancer [33]. To determine if DNA methylation status of the LTR region and the native promoter correlate with their endogenous transcriptional activity, the promoter regions of the LTR-IL-33-expressing cell lines HT115 and LS513, and the native *IL-33*-expressing cell line HUVEC were subjected to bisulfite analysis (Fig 7C). Methylation of the native promoter, which is CpG poor, does not correlate with activity of this promoter. In the case of the LTR region, it is completely unmethylated in HT115 and LS513, the two LTR-positive cell lines and somewhat more methylated in HUVEC, which does not have detectable activity of the LTR. Methylation state of the CpG site just upstream of the LTR TSS correlates most strongly with expression.

### Expression of endogenous LTR-IL-33 protein in colon cancer cell lines

**Fig 8:**
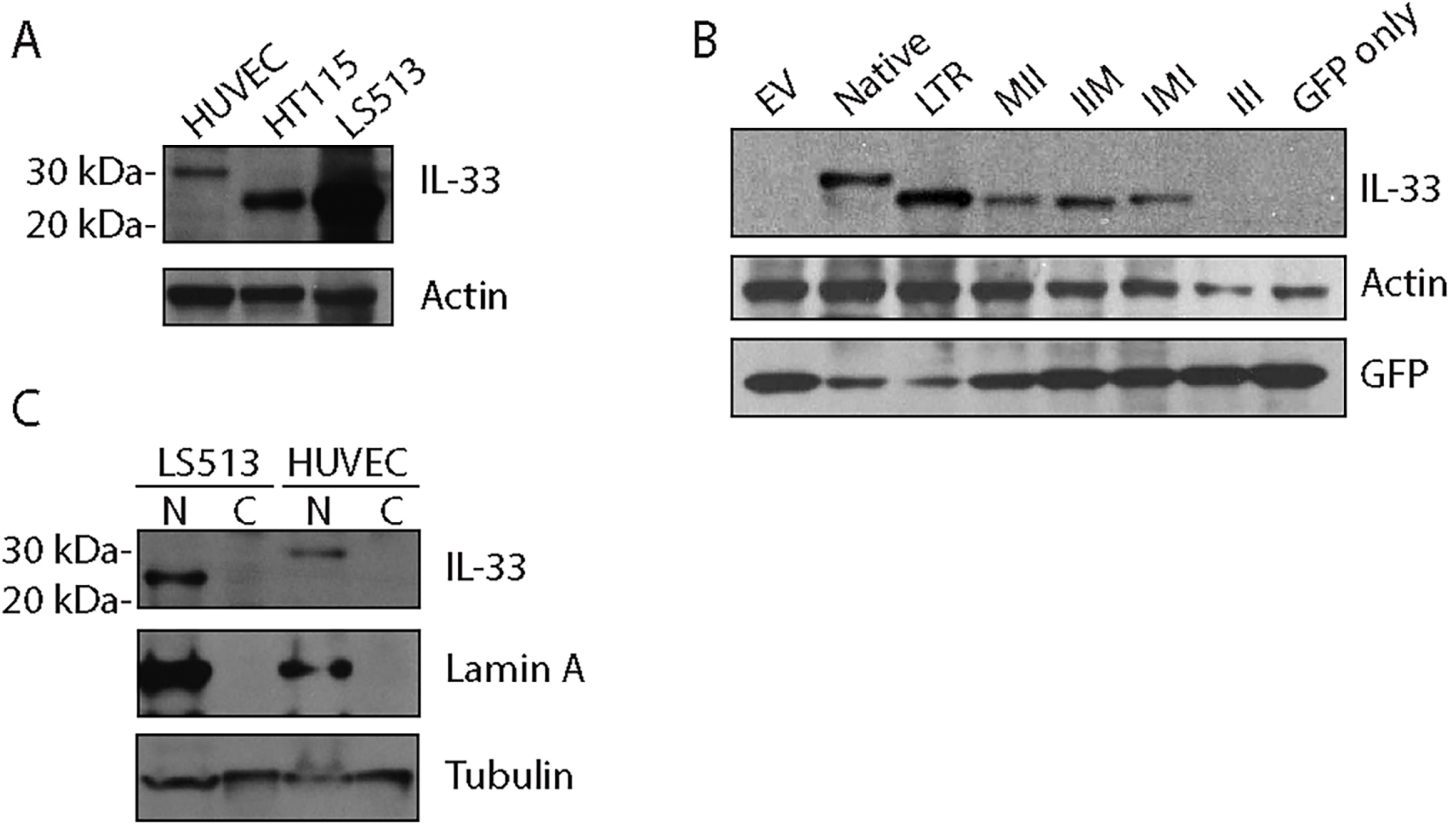
A. Cell lysates were subjected to immunoblotting using an antibody which recognises both the Native and LTR-IL-33 protein isoforms. The predicted molecular weight of LTR-IL-33 is approximately 5kDa less than the native protein. Actin was also assessed as a loading control. B: HEK293T cells were transfected to exogenously express Flag-tagged Native IL-33, LTR-IL-33 or a series of mutants of the LTR-form where two of the three potential translation-initiating Methionines (M) were mutated to Isoleucine (I). The triple III mutant and empty vector control (EV) were also included as negative controls. A GFP expression plasmid was also co-expressed as a transfection control. Total IL-33, GFP and Actin levels were assessed by immunoblotting. C: LS513 or HUVEC cells were cultured to confluence, then lysed and the nuclear and cytosol fractions separated. Lysates were subjected to immunoblotting for IL-33, Lamin A (nuclear marker) and α/β-tubulin as a loading control.

To confirm endogenous expression of LTR-IL-33 protein in the LS513 and HT115 colon cancer cell lines, as predicted by QPCR (Fig 6), western blotting was performed using an IL-33-specific antibody raised against the C-terminal end, able to recognize both the native and predicted LTR-IL-33 isoforms equally (Fig 8A). Due to the truncation of the N-terminal amino acid sequence, the predicted molecular weight of LTR-IL-33 is approximately 5kDa less than the native protein (~26 kDa), hence the two isoforms can be distinguished by standard SDS-PAGE and western blotting techniques. Native IL-33 (~30.7kDa) expression was observed in the positive control HUVEC, as reported previously [85]. LTR-IL-33 protein expression was observed in the colon cancer cell lines positive for the LTR-*IL-33*, LS513 and HT115, in agreement with our QPCR results (Fig 6). Importantly, none of the CRC cell lines were positive for ST2 mRNA expression (S10A Fig), hence do not express a functional IL1R1 signaling complex.

### LTR-IL-33 protein is likely expressed from the first ATG following the LTR

IL-33 is normally retained in the nucleus during homeostasis and interacts with chromatin through a chromatin-binding motif (CBM) localized from amino acids 40–58 (Fig 3B) [101]. It is important to establish which of the possible ATGs is used since this will dictate which amino acids are missing from the LTR IL-33 protein. Importantly, the individual residues essential for chromosome association (CA), within the human IL-33 CBM (aa 40–58), have been identified by mutagenesis: Six residues were required for binding to mitotic chromatin: residues M45, L47, R48, S49, G50 and I53 (Fig 3B) [101], hence usage of the first or second ATG (but not the third) would still produce LTR-IL-33 protein containing the essential CA residues.

None of the three potential in frame ATGs that could be used to translate the LTR-IL-33 isoform (S7 Fig) are in a particularly favorable Kozak context [102]. To determine if these ATGs could initiation translation, we performed site directed mutagenesis to mutate two of the three from methionine to isoleucine in all different combinations and expressed each in the pCMV3Tag3 vector. When exogenously expressed in HEK293T cells (with a 3kDa Flag tag at the C-terminal), each of the three ATG mutants was able to produce protein when the other two were mutated (Fig 8B). Simultaneous mutation of all three ATGs prevented protein expression, as expected. Moreover, in a high-throughput study that evaluated all potential sequences surrounding an ATG from minus 6 to plus 2 for translation efficiency, the sequence context of the first ATG in LTR-IL-33 was found to be the most efficient of the three [103]. Therefore, it is highly likely that the first available ATG is used to translate LTR-IL-33.

### Native and chimeric IL-33 intracellular localization

It has previously been reported that native IL-33, endogenously expressed in HUVEC cells [85], is stored in the nucleus during homeostasis [104], binding to histones H2A-H2B through a chromatin-binding motif (CBM) (amino acids 40–58) (Fig 3B) [101]. As noted above, expression from the LTR results in expression of a chimeric protein missing the 1–41 residues contained in the native second exon. To clarify whether the endogenously expressed LTR-IL-33 isoform was able to localize to the nucleus, LS513 were cultured to confluence, lysed, and the nuclear and cytosolic fractions separated and subjected to Western blotting. HUVEC were also assessed as a native IL-33 positive control. As shown in Fig 8C and S10B Fig, LTR-IL-33 is retained in the nucleus under normal culture conditions, similar to native IL-33. Its ability to retain its nuclear localization is probably due to the retention of the essential chromatin binding residues M45, L47, R48, S49, G50 and I53 [101], as discussed earlier.

### LTR-IL-33 extracellular release studies

**Fig 9:**
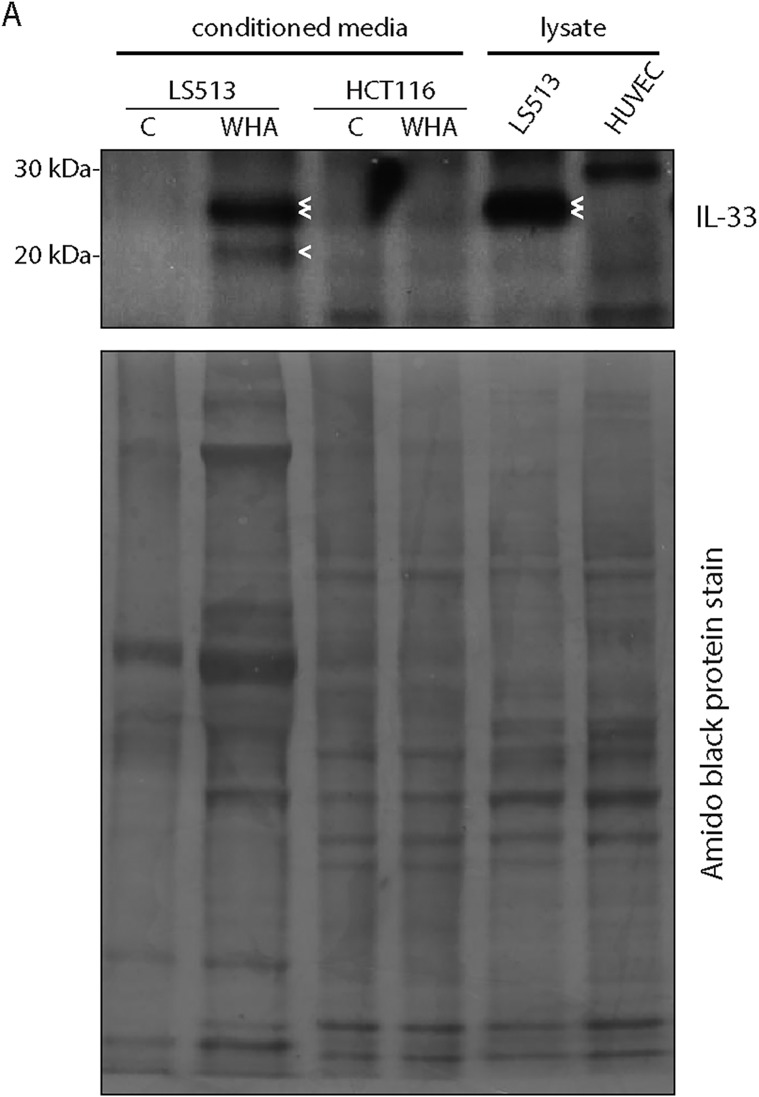
LS513 or HCT116 cells were cultured to confluence, changed into serum-free media and confluent cells “wounded” with pipette tip (wound healing assay, WHA) or not (control, C). After 24h, conditioned media was collected for TCA precipitation of released proteins. Alternatively, LS513 or HUVEC cells were cultured to confluence, and lysates prepared using standard procedures, as a positive control. Media precipitate and lysates were analysed by Western blotting for expression of released or endogenous IL-33 isoforms. Subsequently, the blot membrane was stained with amido black to show protein loading in each lane. Double arrow denotes LTR-IL-33, single arrow denotes cleavage product.

Upon cell damage, native IL-33 is released from cells, providing a damage-associated signal to alert the immune system, hence it is considered an “alarmin” cytokine [67, 68]. Deletion of the N-terminal region responsible for nuclear localization in a mouse model resulted in constitutive release of IL-33 and lethal multi-organ inflammation [97], indicating that nuclear retention is crucial for IL-33 regulation. Extracellular IL-33 can bind to its receptor ST2 on self or neighboring cells, activating receptor-mediated downstream signaling [105]. We chose to assess LTR-IL-33 cellular release in vitro. Previous experiments reported an increase in IL-33 release in vitro following confluent cell layer wounding [85]. Here, we cultured LS153 or the null cell line HCT116 cells to confluence, then changed into serum free media and “wounded”, or not. After 24h, proteins from the conditioned media were precipitated and subjected to immunoblotting, alongside standard LS513 or HUVEC cell culture lysates as a positive control (Fig 9). Full length LTR-IL-33 was present in wounded LS513 conditioned media (and LS513 whole cell lysates) (double arrowhead), but not in unwounded control cell media, similar to findings previously described [85]. A smaller band at approximately 21kDa was also observed in wounded LS513 conditioned media, likely a C-terminal cleavage product, which is not observed in LS513 whole cell lysates (single arrow). This C-terminal cleavage product is similar to the 21kDa C-terminal cleavage product described by Lefrançais et al. [106], which was suggested to be generated by extracellular proteases and be more active compared to full length native IL-33. As expected, conditioned media from the null cell line HCT116 did not show any significant IL-33 expression. HUVEC whole cell lysate lane showed full length native IL-33 as previously.

### Neither native nor LTR-IL-33 appears to function as a transcriptional regulator

**Fig 10:**
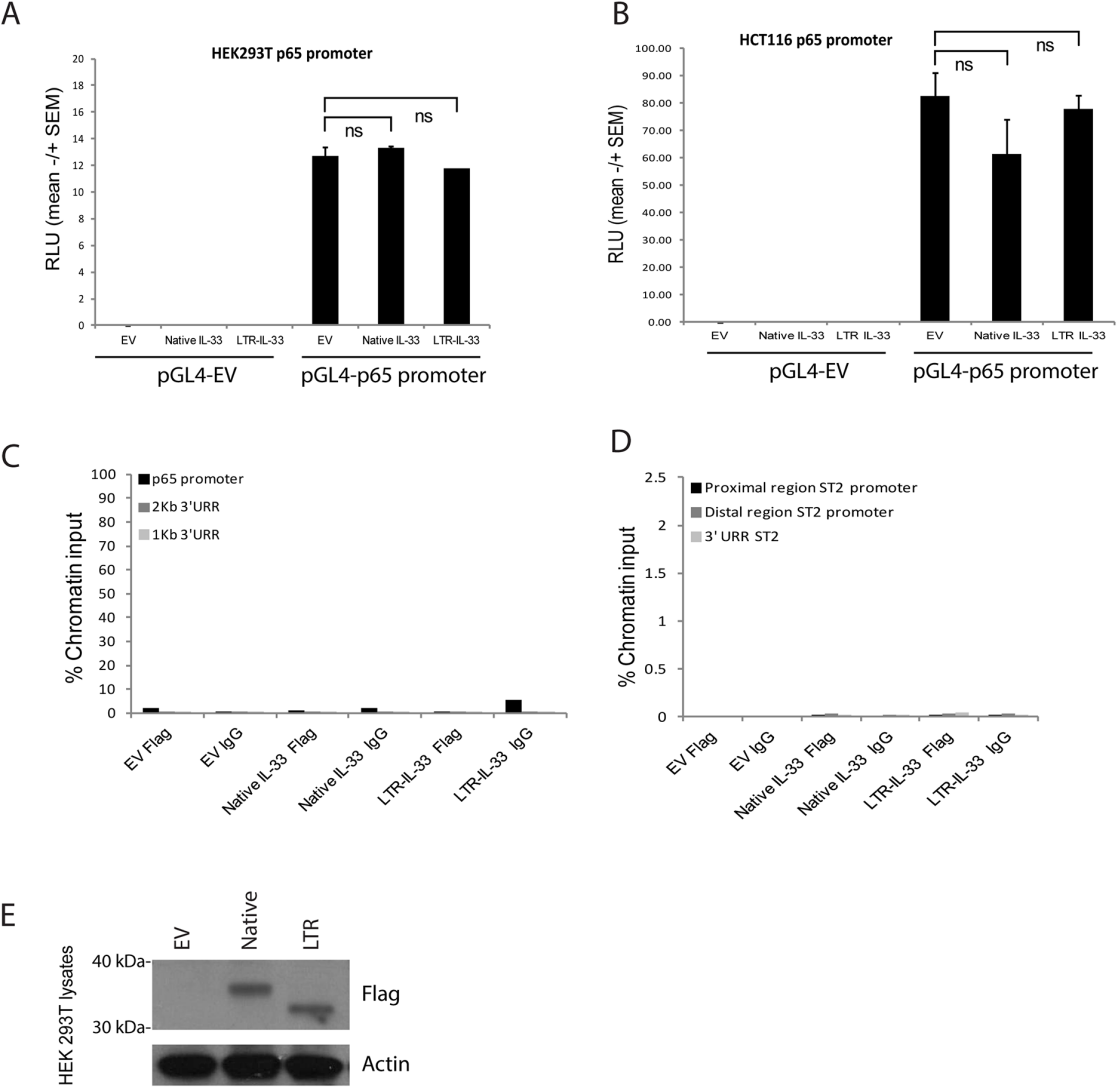
A: HEK 293T or (B) HCT116 cells were transfected to express exogenous Native or LTR-IL-33 (or Empty Flag Vector control) and the full length p65 promoter region in the luciferase construct pGL4 (or the pGL4 Empty Vector control) and Renilla transcription control. After 43h, Relative Luciferase Units were assessed as a measure of p65 promoter activity. C, D: 293T HEK were transfected to express exogenous Native or LTR-IL-33 (or Empty Flag Vector control). 48h later, Chromatin immunoprecipitation was carried out using anti-FLAG antibodies or normal mouse IgG. Genomic DNA was amplified by PCR using primers (C) spanning the p65 promoter region. For the negative control region, genomic DNA was amplified using primers specific to two upstream UnRelated Regions (1kB or 2kB). D: specific to regions (proximal or distal) of the ST2 promoter, or a downstream UnRelated Region. E: HEK293T lysates from cells used in C and D were assessed by Western blotting using antibodies raised against Flag or Actin (loading control). Representative data of 3 independent experiments is shown.

Some studies have suggested that IL-33 can act as a transcriptional regulator through its N-terminal homeodomain-like region (amino acids 1–65) [101, 104]. Specifically, one study reported IL-33 binding to the NF-ĸB *p65* promoter region [82], and another reported IL-33 binding to two regions of the *ST2* (*IL1R1)* promoter [83]. In order to replicate these experiments, Native and LTR-IL-33 were exogenously expressed in the null cell lines HEK293T or HCT116, co-transfected with the p65 promoter region cloned upstream of a luciferase reporter gene. p65 promoter activity was assessed, with no significant changes in p65 promoter activity associated with expression of either IL-33 isoform (Fig 10A and 10B). Binding of either IL-33 isoform to the p65 or ST2 promoter regions was also assessed by ChIP. Again, no evidence of either IL-33 isoform interacting with these promoter regions was observed (Fig 10C and 10D), despite robust exogenous expression of these isoforms (Fig 10E). Finally, endogenous LTR-IL-33 depletion by siRNA in LS513 cells did not affect p65 or TNF alpha (a p65 transcriptional target) mRNA expression in LS513 cells (S10C and S10D Fig). These data are in agreement with a recent large scale proteomics study that found no significant effects on the endothelial proteome upon knock-down of nuclear IL-33, prompting the authors to suggest that IL-33 retention in the nucleus during homeostasis is primarily to regulate its cytokine activity [107].

### Depletion of LTR-IL-33 in vitro does not affect 2D growth of colon cancer cell lines

To assess the role of LTR-IL-33 in colorectal cancer growth under standard in vitro conditions, we treated the LTR-IL-33 positive cell lines, LS513 and HT115, with two previously validated, diverse siRNA targeting IL-33 or a non-silencing control sequence (NSC). Cells were maintained at confluence levels between 90%-100%, as confluency is critical for both LTR-IL-33 and native IL-33 expression [99, 107]. Cells were lysed and subjected to SDS-PAGE and western blotting. LTR-IL-33 expression was significantly decreased following siRNA #1, and more efficiently depleted following siRNA #2 treatment, compared to NSC. Expression of the proliferation marker Proliferating Cell Nuclear Antigen (PCNA) was unchanged (S11A Fig). In addition, exogenous over-expression of Flag-tagged native or LTR-IL-33 did not affect PCNA expression in the IL-33 null CRC cell line HCT116 (S11B and S11C Fig).

### LTR-IL-33 regulates colorectal cancer growth in 3D conditions

**Fig 11:**
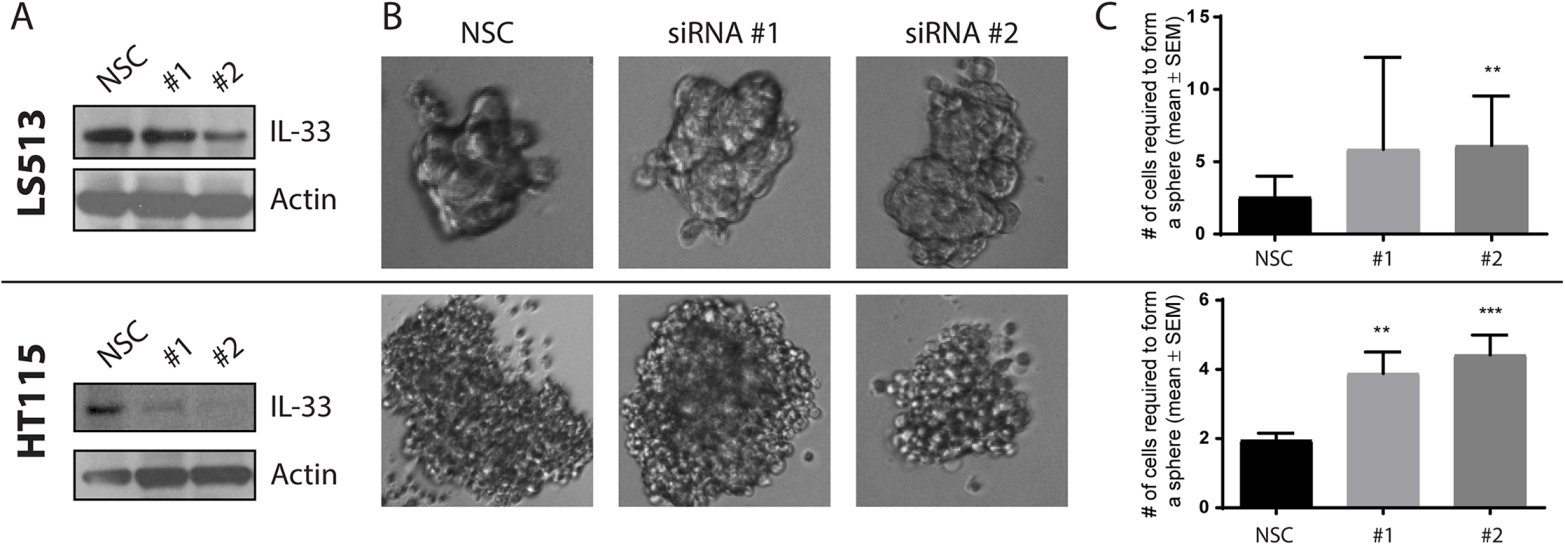
(A) Standard 2D cultures of LS513 or HT115 were treated with siRNA to knockdown expression of IL-33, as confirmed by western blotting (representative images of 3 independent experiments shown). (B) Cells were then seeded in a doubling dilution assay and cultured under colonosphere growth conditions for 7 days. 20x zoom. (C) The number of cells required to form a colonosphere was recorded as a measure of 3D growth (Mean and SEM of 3 experiments shown, T-test: ***, < 0.0001; **, 0.0003).

Since high confluency levels are required for IL-33 expression, standard 2D culture conditions are not suitable for assessing the role of IL-33 in colorectal cancer growth in vitro. Accordingly, we cultured LS513 and HT115 as colonospheres, under 3D, attachment-free serum–free growth conditions [84], since colonosphere formation requires cells to form strong cell–cell interactions to escape anoikis [108]. Untransfected cells from both cell lines were able to grow in suspension as 3D spheres of cells for over 7 days (Fig 11). To assess the role of LTR-IL-33 in colonosphere formation, standard 2D cultures of either cell line were treated with siRNA to knockdown expression of LTR-IL-33, as previously. Transfected cells were then seeded for colonosphere culture in a doubling dilution assay. After 7 days of culture, the number of cells required to form a colonosphere was recorded as a measure of 3D growth. Both LS513 and HT115 showed a significant increase in the number of seeding cells required to form a colonosphere following IL-33 knockdown, suggesting that LTR-IL-33 expression is required for optimal colorectal cancer cell line growth in vitro. Interestingly, when ST2 expression was assessed in both these cell lines, by RT-PCR, neither LS513 nor HT115 were positive for ST2 mRNA expression (S10A Fig), suggesting that these cell lines are null for the IL-33 receptor complex. Hence, the changes observed in 3D following LTR-IL-33 depletion are not due to LTR-IL-33 functioning as a cytokine. These data suggest that LTR-IL-33 may be able to function in a non-cytokine manner, to regulate CRC 3D cell growth, however, the mechanism involved remains to be elucidated. It is theoretically possible that LTR-IL-33 could act as a cytokine through an alternative, currently unidentified, receptor.

## Concluding remarks

We have identified and characterized a novel, shorter isoform of IL-33 driven by an LTR promoter and expressed in a subset of CRC samples. This isoform was also detected in one of five CRC samples by screening RNA-seq from an independent study [109] (S12 Fig). Because highly conserved N-terminal motifs are lacking in this isoform, it is possible that its function may be altered compared to the normal isoform, although we were unable to demonstrate a clear functional difference in this study. We found that the LTR isoform is still able to be retained in the nucleus, but it is possible that loss of the N-terminus could result in changes in function due to changes in protein folding. However, since the LTR-IL-33 C-terminal sequence is unchanged, and the C-terminal alone is sufficient for IL1R1 receptor binding and is a potent activator of IL1R1 signaling [110], it is highly likely that the LTR-IL-33 isoform is able to bind and activate IL1R1 signaling as the native isoform does.

Since the N-terminal homeo-like domain is partly deleted in the LTR-driven isoform, we speculated that its nuclear function might be affected. However, in our experiments we could find no role for native or LTR-IL-33 as a nuclear transcriptional regulator for either of the promoter regions previously identified [82, 83]. It is possible that these previous findings could be cell type specific, since our experiments here exogenously expressed IL-33 in 293T HEK cells, similar to previous studies [104], whereas previous reports [82, 83] used HUVEC and pulmonary arterial endothelial cells, respectively, which both endogenously express IL-33. However, our data concurs with a more recent study carried out in HUVEC cells, which could not identify any reproducible changes in the endothelial cell proteome following knockdown of endogenous nuclear IL-33 [107]. Indeed, nuclear localization of IL-33 is an evolutionary conserved property of the protein observed in all described endogenously expressing cells in both mouse and human tissues [99, 111, 112]. In addition, murine in vivo studies showed that constitutive release of IL-33, following deletion of the N-terminal region responsible for nuclear localization, resulted in lethal multi-organ inflammation, confirming that nuclear retention is crucial for IL-33 regulation [97].

As noted above, previous studies have demonstrated that higher expression of total IL-33 correlates with CRC progression and metastasis, but it should be noted that primers used in these *IL-33* RT-PCR expression assays would have not have distinguished between LTR-IL-33 and the native IL-33 transcript [72, 73]. We show here that expression of both the LTR and native isoforms of *IL-33* combine to result in higher overall levels of *IL-33* in a subset of CRCs. Furthermore, we show that LTR-IL-33 is required for robust 3D growth, in cells lacking the ST2 receptor complex, through a mechanism currently unknown but worth further study. Taken together, these findings suggest that expression of LTR-IL-33 is relevant for colorectal cancer cell proliferation, reflecting its recurrent expression in vivo.

## Supporting information

S1 Table Primers used in this study. (XLSX)

S2 Table Master list of TE-initiated chimeric transcripts. (XLSX)

S3 Table Cancer enriched chimeric transcripts. (XLSX)

S4 Table Normal enriched transcripts. (XLSX)

S1 Text Brief descriptions of four genes shown in Table 1. (PDF)

S1 Fig A) Comparison of numbers of TE-initiated chimeric transcripts between normal and cancer samples based on relaxed thresholds (see Methods). The total number of such transcripts of each TE class was adjusted by their genomic coverage, and also normalized by the expected expression based on all chimeric transcripts in normal samples (the red dotted line). The box plot shows an interquartile range of 50% for each sample group, and outlier samples are shown when the number of chimeric transcripts are beyond one interquartile range from the edge of box. P-values are based T- test. B) Similar plot for the three major ERV classes. C) Total numbers of LTR-initiated chimeric transcripts between normal and cancer samples of each individual patient based on relaxed thresholds. The cancer and normal sample pair of each individual is shown as side-by-side bars in blue and orange, respectively. The height of the bars shows the total number of chimeras in each sample corrected by the library size.
(TIF)

S2 Fig UCSC genome browser views of all CRC samples positive for the LTR-IL-33 isoform. In each case, RNA-seq coverage tracks and resultant assembled transcripts from each CRC sample are shown in red below the Ref-Seq track and above the RepeatMasker track. Green dashed box shows the location of LTR-initiated first exon and a black dashed box shows the native first exon. Note that some samples express both the native and the LTR-promoted isoform.
(TIF)

S3 Fig Representative UCSC genome browser views (hg19) of four genes producing TE-initiated chimeric transcripts in CRC samples and no normal samples. A) INPP4B; B) ACTL8; C) ST8SIA6-AS1; D) MUCL1. In each case, RNA-seq coverage tracks and resultant assembled transcripts from selected CRC samples are shown in red below the Ref-Seq track and above the RepeatMasker track. Direction of transcription is indicated with arrows above the Ref-Seq track. In parts A and B, the region encompassing the TE promoter is enlarged for clarity. The TE promoter/first exon is shown by a dashed green box and is the same as the “normal” annotated promoter for genes in panels B and C. The normal promoter/first exon is shown by dashed black box in panels A and D. For transcript validations, RT-PCR forward primer locations are shown with blue and red stars for the native and TE promoter, respectively. For panels B and C, the native (annotated) and TE promoter are the same. The black stars show locations of the common reverse primers. For panel A, not all of the gene is shown but the common reverse primer is at location chr4:143130126–143130145 (hg19).
(TIF)

S4 Fig UCSC Genome browser views of all CRC samples producing TE-initiated chimeric transcripts for A) *INPP4B* and B) *ST8SIA6-AS1*. In each case, RNA-seq coverage tracks and resultant assembled transcripts from the CRC samples are shown in red below the Ref-Seq track and above the RepeatMasker track. Green dashed boxes show locations of the TE-initiated first exon, which is also the annotated Ref-seq first exon for *ST8SIA6-AS1*. The 5’ portion of *INPP4B* containing the native promoter is not shown in this figure.
(TIF)

S5 Fig UCSC Genome browser views of all CRC samples producing TE-initiated chimeric transcripts for A) *ACTL8* and B) *MUCL1*. In each case, RNA-seq coverage tracks and resultant assembled transcripts from the CRC samples are shown in red below the Ref-Seq track and above the RepeatMasker track. Green dashed boxes show locations of the TE-initiated first exon, which is also the annotated Ref-seq first exon for *ACTL8*.
(TIF)

S6 Fig A) RT-PCR screening of 12 colorectal cancer cell lines to examine expression of native or chimeric gene transcripts. See Table 1 for expression summary. In the case of ACTL8 and ST8SIA6-AS1, the TE promoter is the annotated promoter. GAPDH was also assessed as a housekeeping control. No template control (NTC) was included as a negative control. B) Quantitative RT-PCR screening of 12 colorectal cancer cell lines to examine expression of native IL-33 or (chimeric) LTR-IL-33 gene transcripts. As reflected in Table 1, the three cell lines LoVo, HT115 and LS513 were routinely positive for LTR-IL-33 (above the threshold of 1).
(TIF)

S7 Fig A) cDNA from HT115 cells was subjected to PCR using primer sequences from MSTD-LTR (IL33_LTR_F) and final IL-33 exon (IL33_tot_R) (see S1 Table for details), giving a single 1100bp amplicon, as expected. Amplicon was sequenced and LTR-IL-33 sequence was confirmed. B) Sequence and amino acid translation of the LTR-IL-33 cDNA cloned using primers highlighted in yellow from HT-115 cells. Three potential methionine start codons are highlighted in green. C) Genome browser screenshot to show placement of primers (IL33_LTR_F and IL33_tot_R, circled in red) used to amplify full length LTR-IL-33 cDNA for subsequent sequencing analysis.
(TIF)

S8 Fig A) To confirm sequence of chimeric 5′ ends, 5′ RACE was performed and run on an agarose gel. The major band of PCR-amplified 5′ end transcripts (boxed in red) were cloned into pGEM T vector and sequenced. B) MST2D LTR region. Blue sections show the LTR and green sections are two antisense Alu elements inserted within the LTR. The major TSS site identified in 12 of 14 5’RACE clones is shown with an asterisk above the sequence and the putative TATA motif is boxed. The splice donor site (SD) is indicated with a vertical line. Primers used to clone the LTR for promoter assays are shown with underlined arrows. The Bgl2 site used to clone the intermediate-sized promoter construct is underlined in italics. Five CpG sites assayed by bisulfite sequencing are bolded and underlined.
(TIF)

S9 Fig Short and Long forms of the MSTD-LTR promoter luciferase constructs were transfected into (A) HT115 (B) HCT116 or (C) HEK 293T cells and activity assessed by relative luciferase assay. Data representative of 3 independent experiments is shown. (D) Various cell lysates were subjected to immnoblotting using an antibody which recognises both the Native and LTR-IL-33 protein isoforms. Actin was also assessed as a loading control.
(TIF)

S10 Fig A) RT-PCR screening of various cell lines (and primary Human Tonsilitis lymphocyte fraction, HTL) to examine expression of ST2 mRNA. GAPDH was assessed as a housekeeping control. No template control (NTC) was included as a negative control. B) HT115 cells were cultured to confluence, then lysed and the nuclear and cytosol fractions separated. Lysates were subjected to immunoblotting for IL-33 with blot amido black staining shown as a loading control. C) LS513 cells were transiently transfected with siRNA targeting IL-33. Depletion of IL-33 mRNA was assessed by quantitative RT-PCR. D) mRNA samples were subsequently assessed for p65 and TNF alpha mRNA expression by semi-quantitative RT-PCR. Data shown is representative of 2 independent experiments.
(TIF)

S11 Fig A) LS513 cells were transfected with two diverse siRNA targeting IL-33 or a non-silencing control sequence. Cells were lysed and subjected to SDS-PAGE and western blotting with antibodies raised against IL-33, PCNA or actin as a loading control. B) HCT116 were transiently transfected, in triplicate, to exogenously express Flag tagged Native-IL-33, LTR-IL-33 or EV control. After 48h, cells were lysed and assessed by western blot for PCNA expression. IL-33 exogenous expression was also confirmed, and actin assessed as a loading control. C) densitometry data was analyzed by T-test. Representative data of 2 independent experiments are shown.
(TIF)

S12 Fig UCSC genome brower view (hg19) of the IL-33 region showing RNA-seq coverage from an independent cohort of 5 CRC (C1-C5) samples and matched normal colon (N1-N5)[109]. Cuffink assembled transcripts for the C5 CRC sample, which is positive for the LTR-initiated isoform, are shown below the coverage track. This CRC is positive for both the native promoter (localized by black dashed box) and the LTR promoter (localized by green dashed box).
(TIF)
