# Supplementary material for: A novel isoform of IL-33 revealed by screening for transposable element promoted genes in human colorectal cancer
Source: PLoS One. 2017 Jul 17;12(7):e0180659. doi: 10.1371/journal.pone.0180659 (PMC5513427; doi:10.1371/journal.pone.0180659)
Supplement: S1 Text — (PDF) [file pone.0180659.s005.pdf]

Text S1: Brief descriptions of four genes shown in Table 1.

INPP4B: Inositol polyphosphate 4-phosphatase type II (INPP4B) is one of several enzymes that maintain a critical balance of phosphoinositides in cells. Several studies have shown that INPP4B acts as a tumor suppressor in ovarian, prostate and basal-type breast cancers [1-3]. However, a number of recent studies indicate that INPP4B plays an oncogenic role in luminal-type breast cancer[4], acute myeloid leukemia[5,6], melanoma[7] and colon cancer[8]. We identified an antisense L1PA14 element that appears to be serving as a promoter for this gene in a subset of CRCs that, if translated, would produce a protein identical to the native form since the native ATG is in a downstream exon (Fig. S3A). Several ESTs and mRNAs annotated in the UCSC Genome Browser initiate in the L1 element but no previous publications on *INPP4B* have addressed alternative promoter usage.

ACTL8: The actin-like 8 (*ACTL8*) gene is a marker for basal-type breast cancer[9] and has been characterized as a cancer-testis (CT) antigen, which are genes with expression restricted primarily to normal testis and cancer cells[10]. The annotated promoter for this gene maps to an LTR41 ERV element and our screen detected it in ~10% of CRC RNA-Seq samples (Fig. S3B).

ST8SIA6-AS1: This lncRNA is antisense to, and is contained within, the gene encoding ST8 Alpha-N-Acetyl-Neuraminide Alpha-2,8-Sialyltransferase 6 (*ST8SIA6*). Its annotated promoter is a MER48 LTR and we detected its expression in five CRC samples (Fig. S3C). A recent study on lncRNA expression in the HER2-enriched subtype of breast cancer reported that *ST8SIA6-AS1* is highly dysregulated in this disease[11].

MUCL1: Several studies have reported that this mucin-like 1 gene, also termed *SBEM*, is a marker for aggressive breast cancer[12-14]. A recent study demonstrated that MUCL1 acts as an oncogene by positively regulating cell growth in HER2-positive breast cancers[14]. We found that an upstream MER31 ERV promotes transcription of this gene in a small percentage of CRC samples by splicing into the second exon (Fig. S3D). However, since the ATG is in the first exon and there are no other in frame ATGs, the chimeric form is unlikely to be translated and its significance is unclear. An EST and a cluster of CAGE tags from the Fantom 5 project (http://fantom.gsc.riken.jp/5/) also map to this MER31 ERV.

Supplementary References

1. Fedele CG, Ooms LM, Ho M, Vieusseux J, O'Toole SA, et al. (2010) Inositol polyphosphate 4-phosphatase II regulates PI3K/Akt signaling and is lost in human basal-like breast cancers. Proceedings of the National Academy of Sciences 107: 22231-22236.
2. Rynkiewicz NK, Fedele CG, Chiam K, Gupta R, Kench JG, et al. (2015) INPP4B is highly expressed in prostate intermediate cells and its loss of expression in prostate carcinoma predicts for recurrence and poor long term survival. The Prostate 75: 92-102.
3. Agoulnik IU, Hodgson MC, Bowden WA, Ittmann MM (2011) INPP4B: the New Kid on the PI3K Block.
4. Gasser Jessica A, Inuzuka H, Lau Alan W, Wei W, Beroukhim R, et al. (2014) SGK3 Mediates INPP4B-Dependent PI3K Signaling in Breast Cancer. Molecular Cell 56: 595-607.
5. Rijal S, Fleming S, Cummings N, Rynkiewicz NK, Ooms LM, et al. (2015) Inositol polyphosphate 4-phosphatase II (INPP4B) is associated with chemoresistance and poor outcome in AML. Blood 125: 2815-2824.
6. Dzneladze I, He R, Woolley JF, Son MH, Sharobim MH, et al. (2015) INPP4B overexpression is associated with poor clinical outcome and therapy resistance in acute myeloid leukemia. Leukemia 29: 1485-1495.
7. Chi MN, Guo ST, Wilmott JS, Guo XY, Yan XG, et al. (2015) INPP4B is upregulated and functions as an oncogenic driver through SGK3 in a subset of melanomas. Oncotarget 6: 39891-39907.
8. Guo ST, Chi MN, Yang RH, Guo XY, Zan LK, et al. (2015) INPP4B is an oncogenic regulator in human colon cancer. Oncogene 35: 3049-3061.
9. Yao J, Caballero OL, Yung WKA, Weinstein JN, Riggins GJ, et al. (2014) Tumor Subtype-Specific Cancer–Testis Antigens as Potential Biomarkers and Immunotherapeutic Targets for Cancers. Cancer Immunology Research 2: 371-379.
10. Whitehurst AW (2014) Cause and Consequence of Cancer/Testis Antigen Activation in Cancer. Annual Review of Pharmacology and Toxicology 54: 251-272.
11. Yang F, Lyu S, Dong S, Liu Y, Zhang X, et al. (2016) Expression profile analysis of long noncoding RNA in HER-2-enriched subtype breast cancer by next-generation sequencing and bioinformatics. OncoTargets and therapy 9: 761-772.
12. Miksicek RJ, Myal Y, Watson PH, Walker C, Murphy LC, et al. (2002) Identification of a Novel Breast- and Salivary Gland-specific, Mucin-like Gene Strongly Expressed in Normal and Tumor Human Mammary Epithelium. Cancer Research 62: 2736-2740.
13. Liu Z-Z, Xie X-D, Qu S-X, Zheng Z-D, Wang Y-K (2010) Small breast epithelial mucin (SBEM) has the potential to be a marker for predicting hematogenous micrometastasis and response to neoadjuvant chemotherapy in breast cancer. Clinical & Experimental Metastasis 27: 251-259.
14. Conley SJ, Bosco EE, Tice DA, Hollingsworth RE, Herbst R, et al. (2016) HER2 drives Mucin-like 1 to control proliferation in breast cancer cells. Oncogene 35: 4225-4234.
