# Supplementary figures and images for: A novel isoform of IL-33 revealed by screening for transposable element promoted genes in human colorectal cancer
Source: PLoS One. 2017 Jul 17;12(7):e0180659. doi: 10.1371/journal.pone.0180659 (PMC5513427; doi:10.1371/journal.pone.0180659)

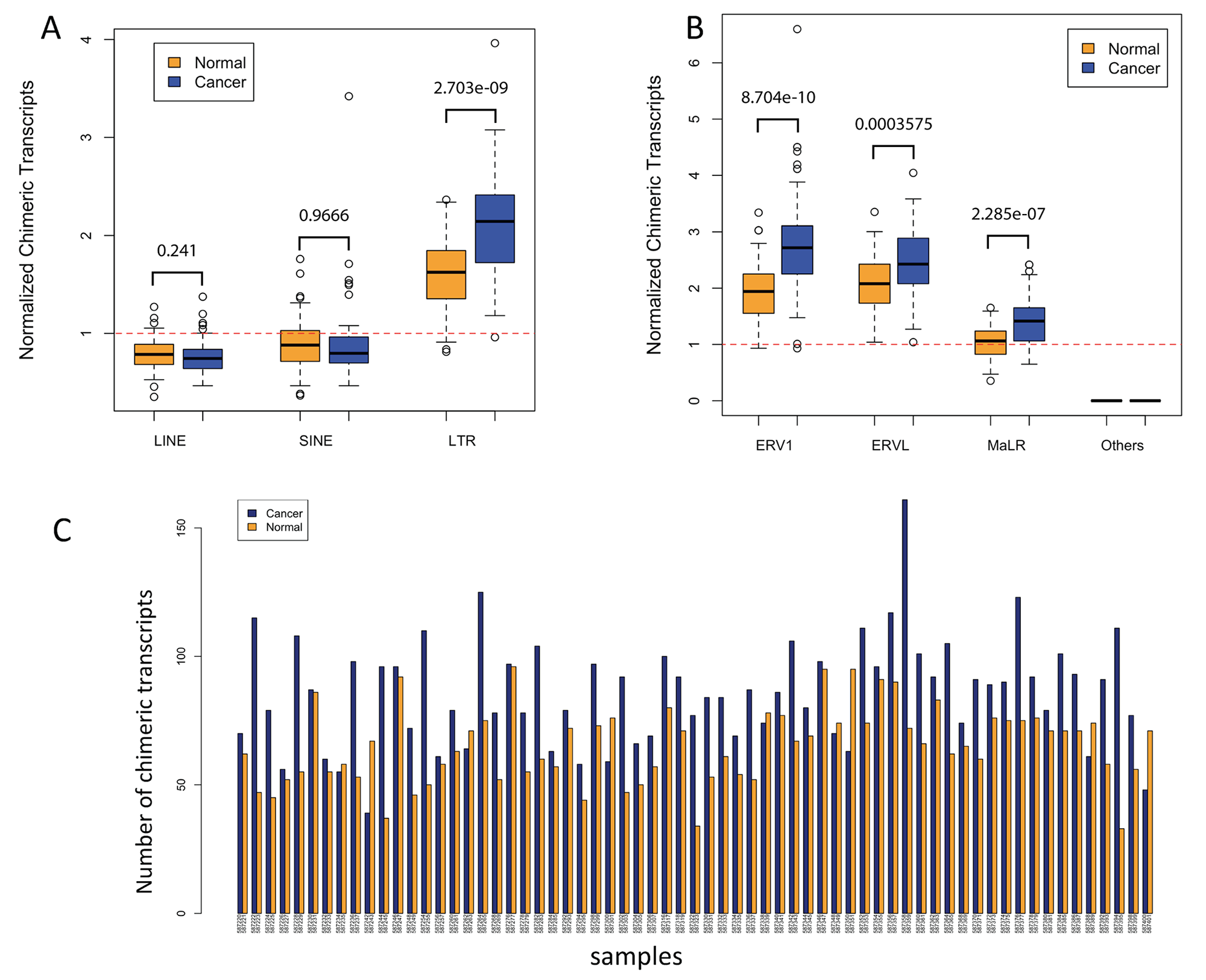

Supplement: S1 Fig — A) Comparison of numbers of TE-initiated chimeric transcripts between normal and cancer samples based on relaxed thresholds (see Methods). The total number of such transcripts of each TE class was adjusted by their genomic coverage, and also normalized by the expected expression based on all chimeric transcripts in normal samples (the red dotted line). The box plot shows an interquartile range of 50% for each sample group, and outlier samples are shown when the number of chimeric transcripts are beyond one interquartile range from the edge of box. P-values are based T- test. B) Similar plot for the three major ERV classes. C) Total numbers of LTR-initiated chimeric transcripts between normal and cancer samples of each individual patient based on relaxed thresholds. The cancer and normal sample pair of each individual is shown as side-by-side bars in blue and orange, respectively. The height of the bars shows the total number of chimeras in each sample corrected by the library size. (TIF) [file pone.0180659.s006.tif]

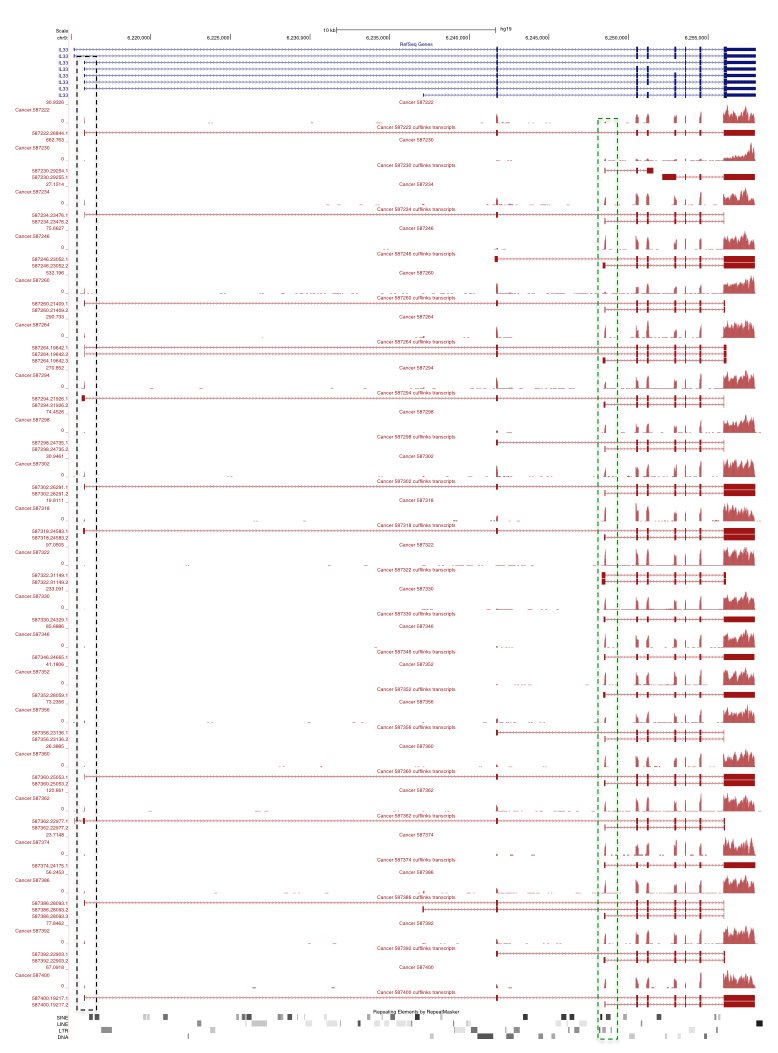

Supplement: S2 Fig — In each case, RNA-seq coverage tracks and resultant assembled transcripts from each CRC sample are shown in red below the Ref-Seq track and above the RepeatMasker track. Green dashed box shows the location of LTR-initiated first exon and a black dashed box shows the native first exon. Note that some samples express both the native and the LTR-promoted isoform. (TIF) [file pone.0180659.s007.tif]

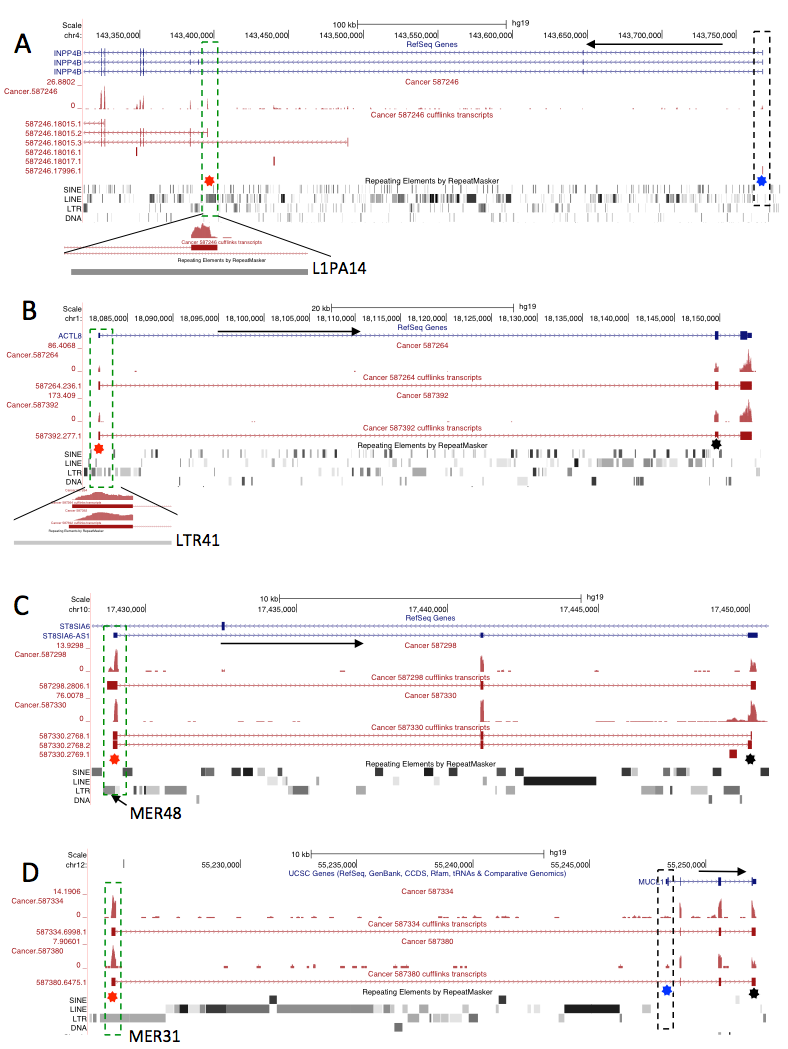

Supplement: S3 Fig — A) INPP4B; B) ACTL8; C) ST8SIA6-AS1; D) MUCL1. In each case, RNA-seq coverage tracks and resultant assembled transcripts from selected CRC samples are shown in red below the Ref-Seq track and above the RepeatMasker track. Direction of transcription is indicated with arrows above the Ref-Seq track. In parts A and B, the region encompassing the TE promoter is enlarged for clarity. The TE promoter/first exon is shown by a dashed green box and is the same as the “normal” annotated promoter for genes in panels B and C. The normal promoter/first exon is shown by dashed black box in panels A and D. For transcript validations, RT-PCR forward primer locations are shown with blue and red stars for the native and TE promoter, respectively. For panels B and C, the native (annotated) and TE promoter are the same. The black stars show locations of the common reverse primers. For panel A, not all of the gene is shown but the common reverse primer is at location chr4:143130126–143130145 (hg19). (TIF) [file pone.0180659.s008.tif]

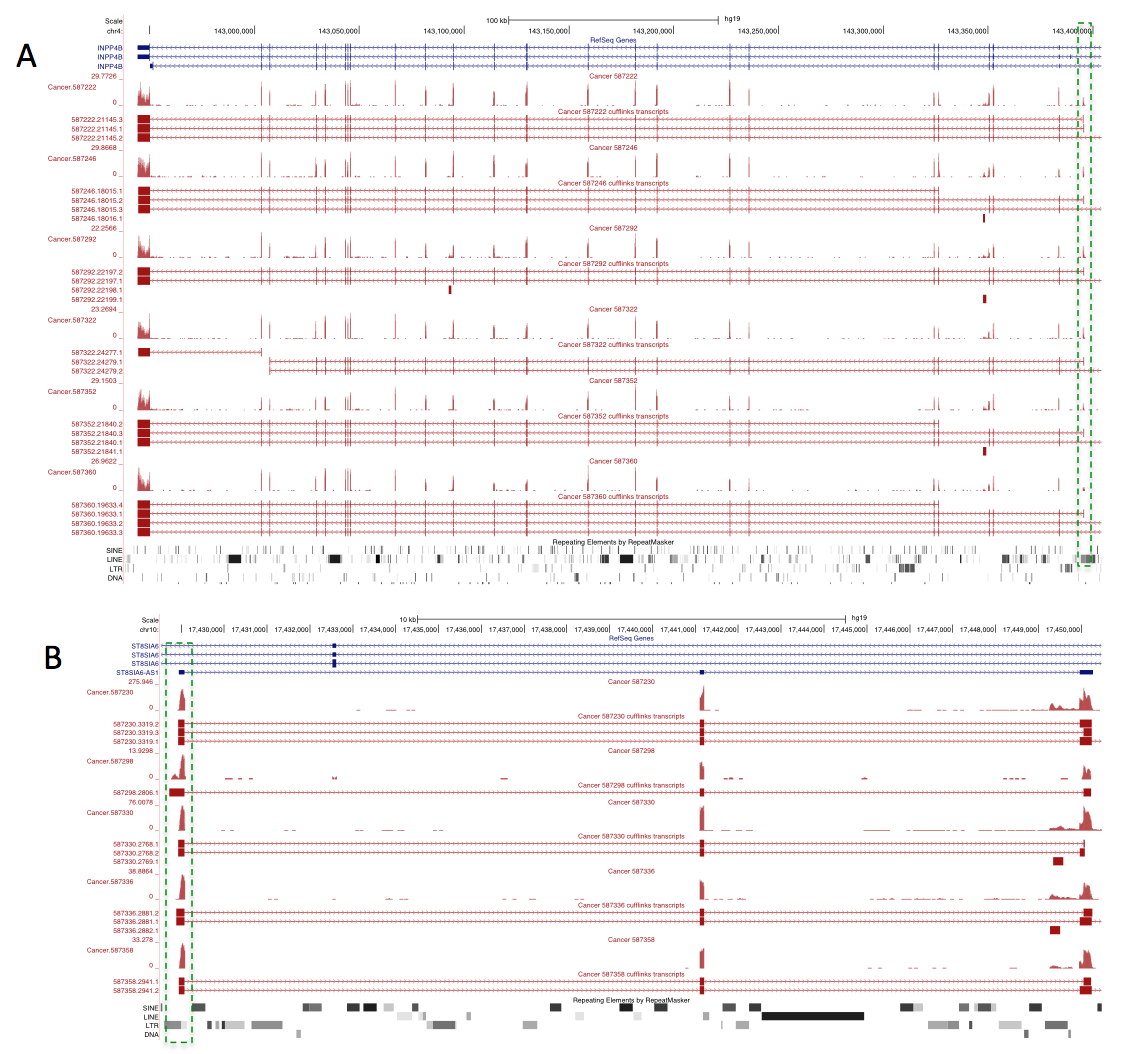

Supplement: S4 Fig — UCSC Genome browser views of all CRC samples producing TE-initiated chimeric transcripts for A) INPP4B and B) ST8SIA6-AS1. In each case, RNA-seq coverage tracks and resultant assembled transcripts from the CRC samples are shown in red below the Ref-Seq track and above the RepeatMasker track. Green dashed boxes show locations of the TE-initiated first exon, which is also the annotated Ref-seq first exon for ST8SIA6-AS1. The 5’ portion of INPP4B containing the native promoter is not shown in this figure. (TIF) [file pone.0180659.s009.tif]

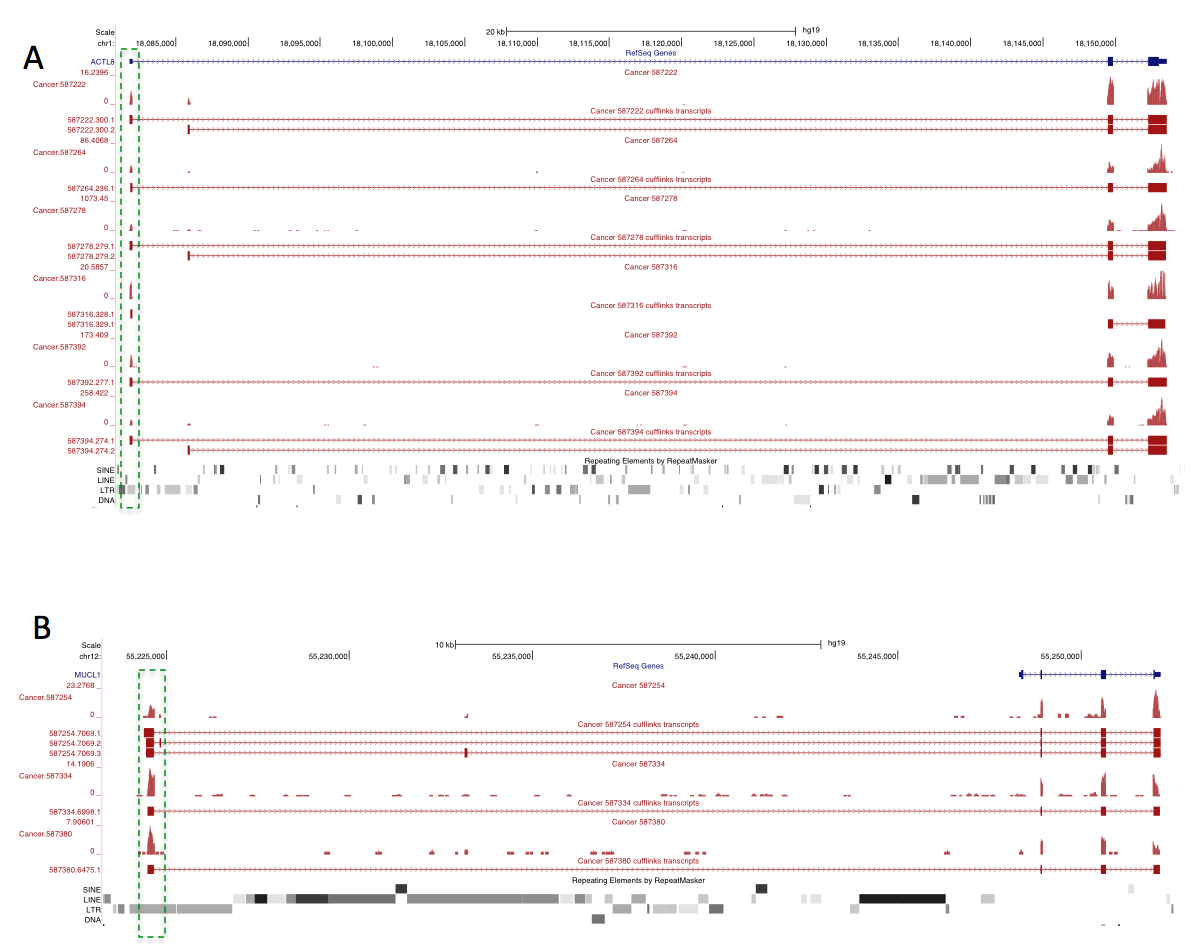

Supplement: S5 Fig — UCSC Genome browser views of all CRC samples producing TE-initiated chimeric transcripts for A) ACTL8 and B) MUCL1. In each case, RNA-seq coverage tracks and resultant assembled transcripts from the CRC samples are shown in red below the Ref-Seq track and above the RepeatMasker track. Green dashed boxes show locations of the TE-initiated first exon, which is also the annotated Ref-seq first exon for ACTL8. (TIF) [file pone.0180659.s010.tif]

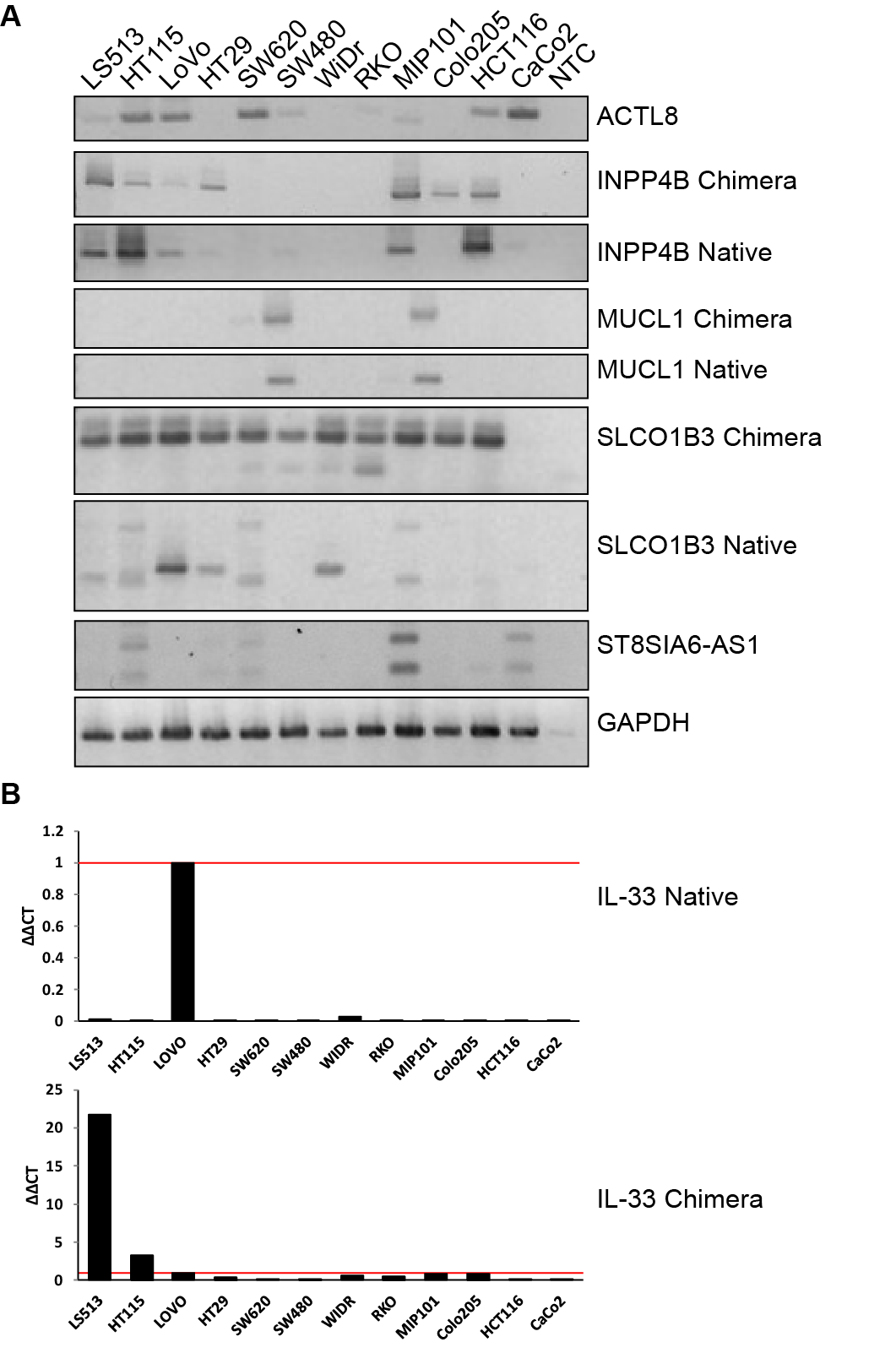

Supplement: S6 Fig — A) RT-PCR screening of 12 colorectal cancer cell lines to examine expression of native or chimeric gene transcripts. See Table 1 for expression summary. In the case of ACTL8 and ST8SIA6-AS1, the TE promoter is the annotated promoter. GAPDH was also assessed as a housekeeping control. No template control (NTC) was included as a negative control. B) Quantitative RT-PCR screening of 12 colorectal cancer cell lines to examine expression of native IL-33 or (chimeric) LTR-IL-33 gene transcripts. As reflected in Table 1, the three cell lines LoVo, HT115 and LS513 were routinely positive for LTR-IL-33 (above the threshold of 1). (TIF) [file pone.0180659.s011.tif]

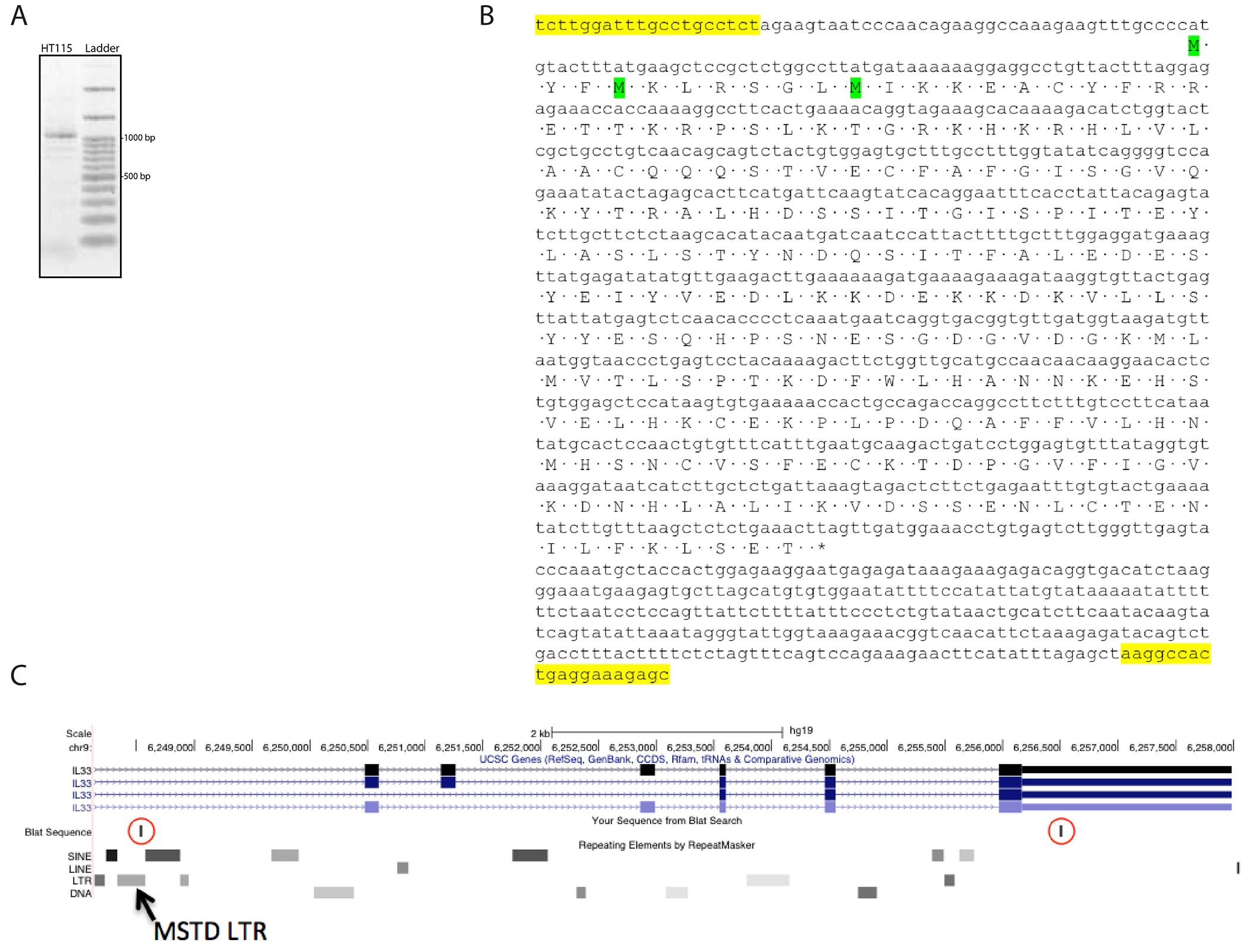

Supplement: S7 Fig — A) cDNA from HT115 cells was subjected to PCR using primer sequences from MSTD-LTR (IL33_LTR_F) and final IL-33 exon (IL33_tot_R) (see S1 Table for details), giving a single 1100bp amplicon, as expected. Amplicon was sequenced and LTR-IL-33 sequence was confirmed. B) Sequence and amino acid translation of the LTR-IL-33 cDNA cloned using primers highlighted in yellow from HT-115 cells. Three potential methionine start codons are highlighted in green. C) Genome browser screenshot to show placement of primers (IL33_LTR_F and IL33_tot_R, circled in red) used to amplify full length LTR-IL-33 cDNA for subsequent sequencing analysis. (TIF) [file pone.0180659.s012.tif]

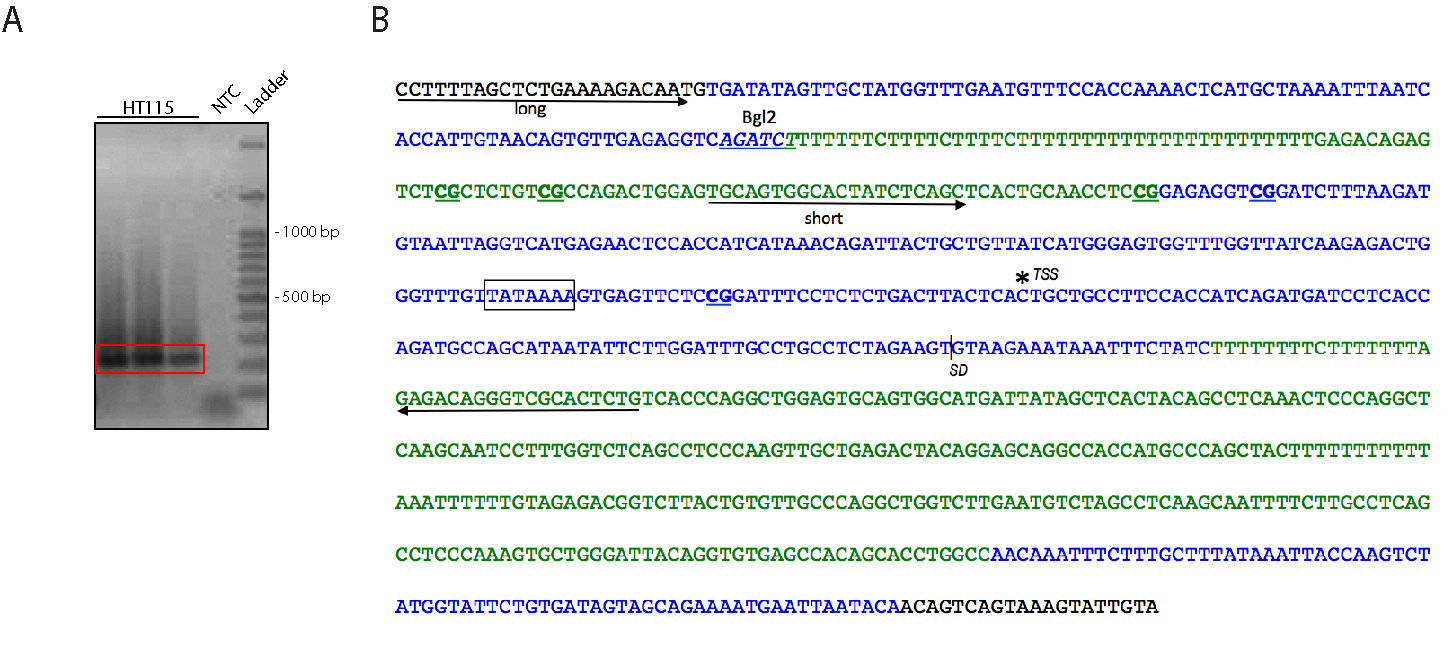

Supplement: S8 Fig — A) To confirm sequence of chimeric 5′ ends, 5′ RACE was performed and run on an agarose gel. The major band of PCR-amplified 5′ end transcripts (boxed in red) were cloned into pGEM T vector and sequenced. B) MST2D LTR region. Blue sections show the LTR and green sections are two antisense Alu elements inserted within the LTR. The major TSS site identified in 12 of 14 5’RACE clones is shown with an asterisk above the sequence and the putative TATA motif is boxed. The splice donor site (SD) is indicated with a vertical line. Primers used to clone the LTR for promoter assays are shown with underlined arrows. The Bgl2 site used to clone the intermediate-sized promoter construct is underlined in italics. Five CpG sites assayed by bisulfite sequencing are bolded and underlined. (TIF) [file pone.0180659.s013.tif]

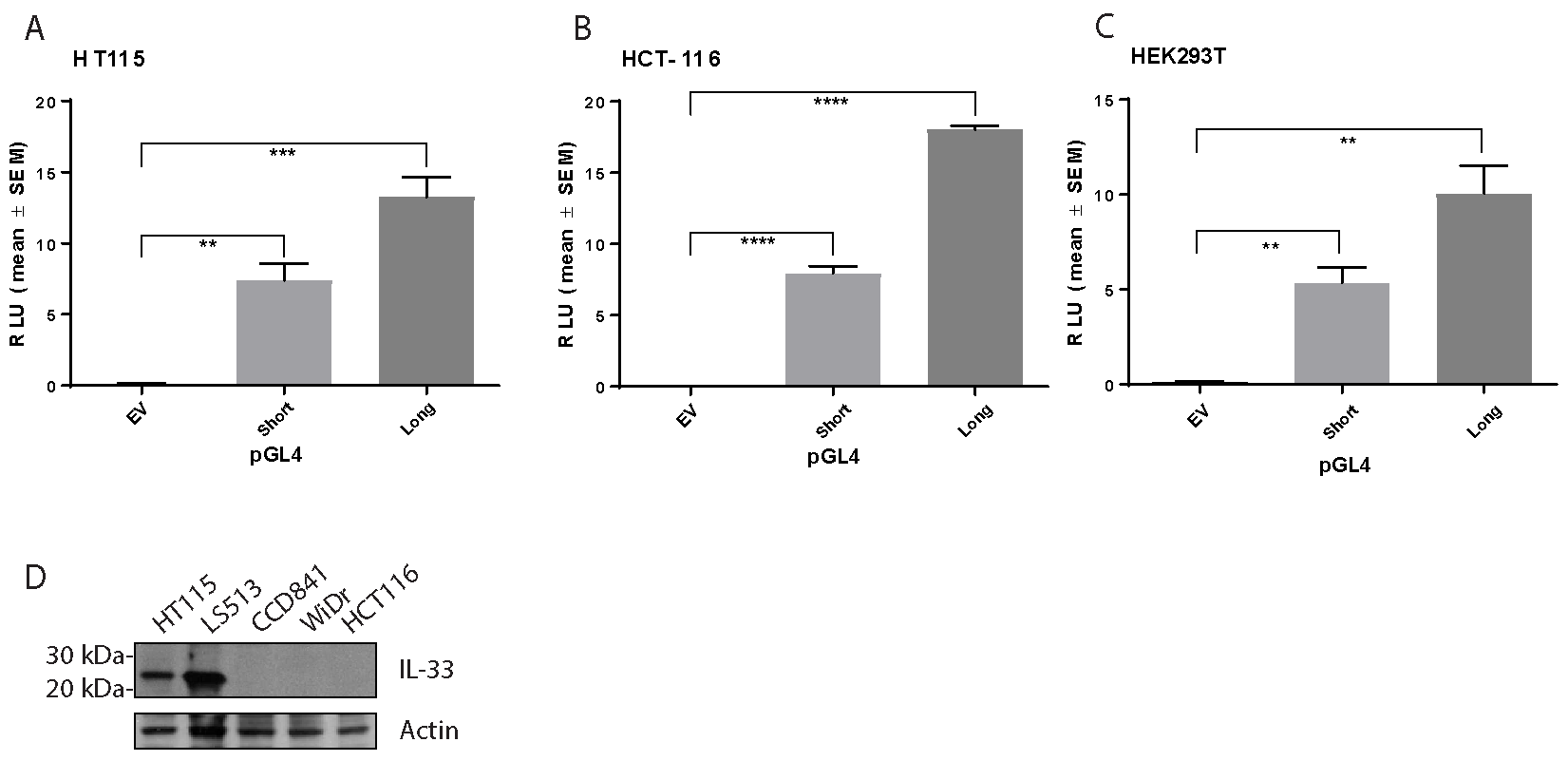

Supplement: S9 Fig — Short and Long forms of the MSTD-LTR promoter luciferase constructs were transfected into (A) HT115 (B) HCT116 or (C) HEK 293T cells and activity assessed by relative luciferase assay. Data representative of 3 independent experiments is shown. (D) Various cell lysates were subjected to immnoblotting using an antibody which recognises both the Native and LTR-IL-33 protein isoforms. Actin was also assessed as a loading control. (TIF) [file pone.0180659.s014.tif]

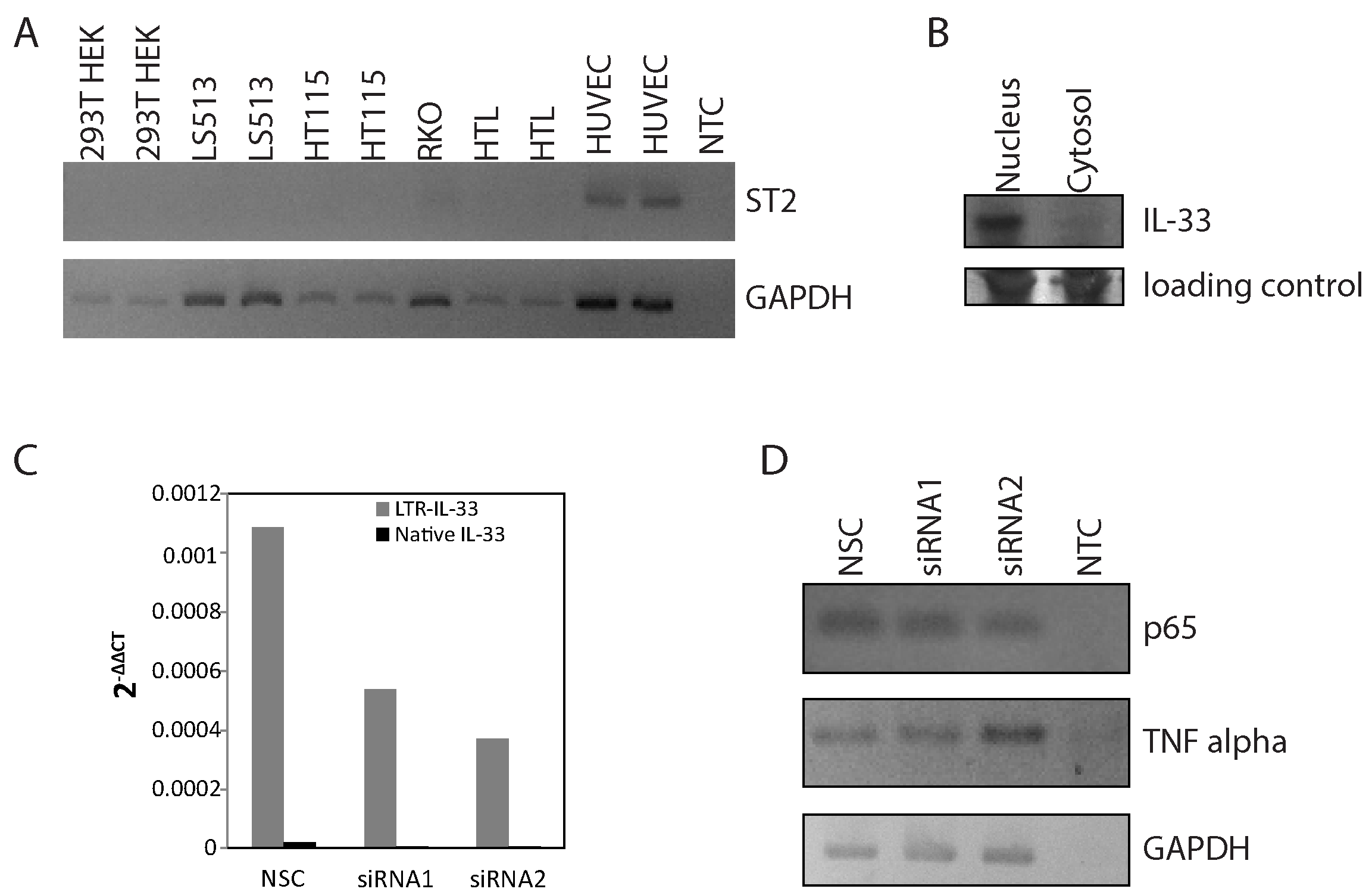

Supplement: S10 Fig — A) RT-PCR screening of various cell lines (and primary Human Tonsilitis lymphocyte fraction, HTL) to examine expression of ST2 mRNA. GAPDH was assessed as a housekeeping control. No template control (NTC) was included as a negative control. B) HT115 cells were cultured to confluence, then lysed and the nuclear and cytosol fractions separated. Lysates were subjected to immunoblotting for IL-33 with blot amido black staining shown as a loading control. C) LS513 cells were transiently transfected with siRNA targeting IL-33. Depletion of IL-33 mRNA was assessed by quantitative RT-PCR. D) mRNA samples were subsequently assessed for p65 and TNF alpha mRNA expression by semi-quantitative RT-PCR. Data shown is representative of 2 independent experiments. (TIF) [file pone.0180659.s015.tif]

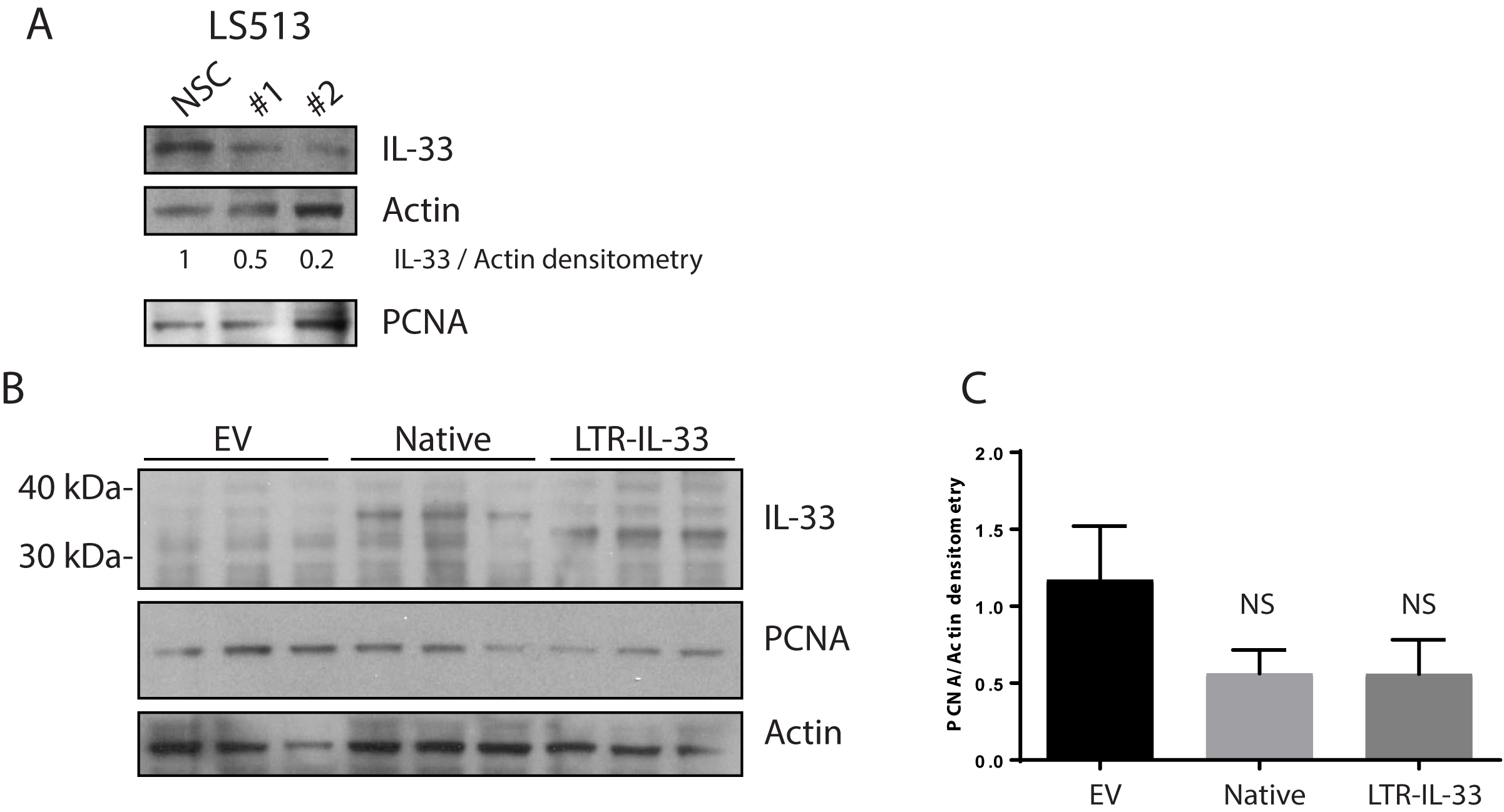

Supplement: S11 Fig — A) LS513 cells were transfected with two diverse siRNA targeting IL-33 or a non-silencing control sequence. Cells were lysed and subjected to SDS-PAGE and western blotting with antibodies raised against IL-33, PCNA or actin as a loading control. B) HCT116 were transiently transfected, in triplicate, to exogenously express Flag tagged Native-IL-33, LTR-IL-33 or EV control. After 48h, cells were lysed and assessed by western blot for PCNA expression. IL-33 exogenous expression was also confirmed, and actin assessed as a loading control. C) densitometry data was analyzed by T-test. Representative data of 2 independent experiments are shown. (TIF) [file pone.0180659.s016.tif]

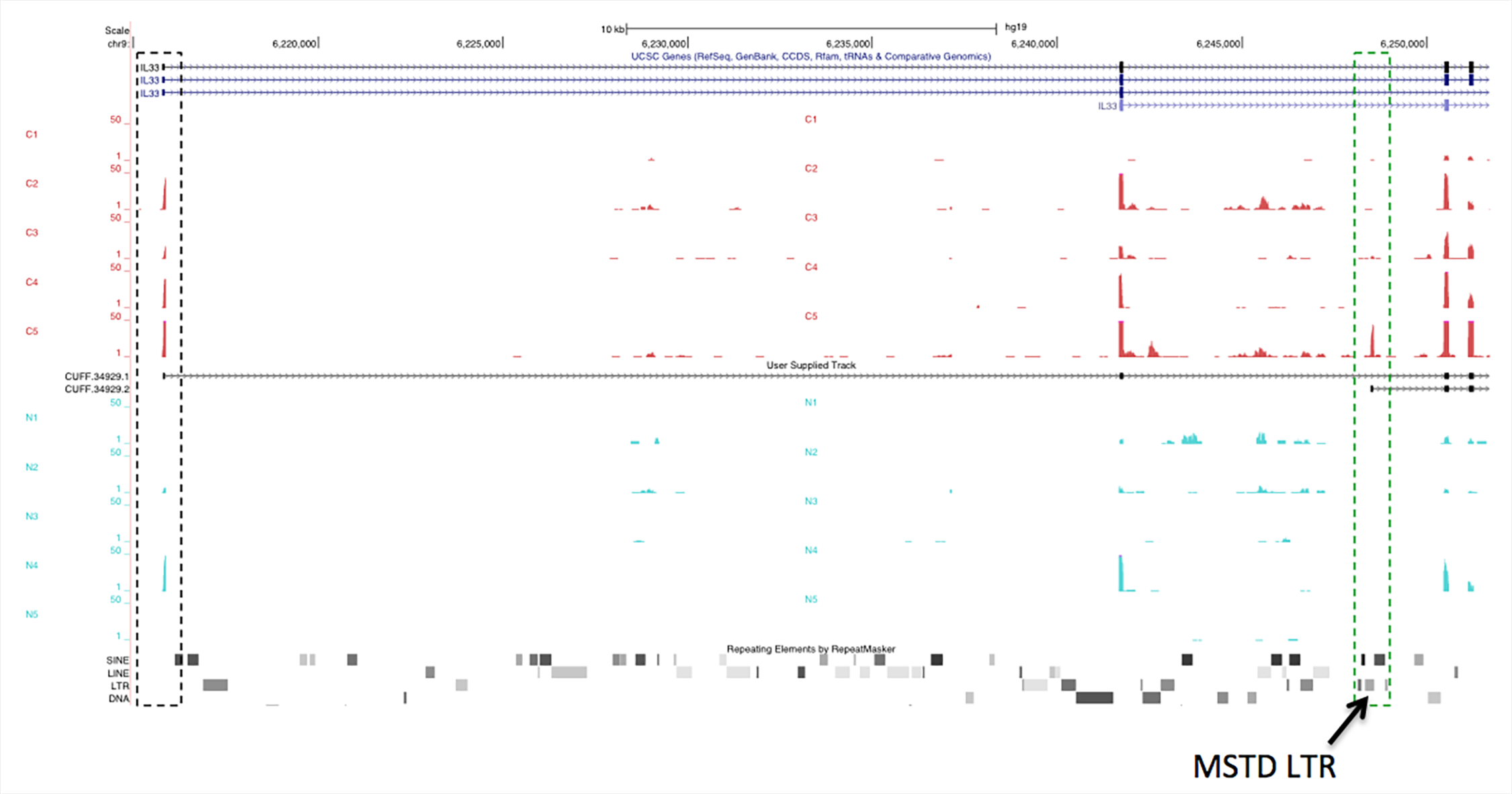

Supplement: S12 Fig — Cuffink assembled transcripts for the C5 CRC sample, which is positive for the LTR-initiated isoform, are shown below the coverage track. This CRC is positive for both the native promoter (localized by black dashed box) and the LTR promoter (localized by green dashed box). (TIF) [file pone.0180659.s017.tif]
